# Development and content validity of Diet Quality Index for Athletes (DQI-Athletes)

**DOI:** 10.1186/s40795-026-01282-8

**Published:** 2026-09-21

**Authors:** Neelam M. Rathod, Suneeta S. Chandorkar

**Affiliations:** Department of Foods and Nutrition, Faculty of Family and Community Sciences, The Maharaja Sayajirao University of Baroda, Vadodara, Gujarat India

**Keywords:** Diet Quality Index, Athletes, Content validity, Dietary assessment, Sports nutrition

## Abstract

**Introduction:**

Dietary recommendations for athletes vary according to the type of sport, and the use of uniform assessment criteria may not accurately capture diet quality of athletes from different sport disciplines and weight categories. For example, applying the same guidelines to power and skill sport athletes may lead to inaccurate scoring, similar to the limitations observed with population-based diet quality indices. Therefore, this study aimed to develop a Diet Quality Index for Athletes(DQI-Athletes) and establish its content validity for the Indian athletic population.

**Methodology:**

DQI-Athletes included four component types: adequacy,moderation, ratio, and dietary behaviours. Total score of DQI-Athletes ranged from 0 to 100. A total of 108 athletes(≥ 18 years; 52 females), currently in the training phase and representing diverse sports disciplines, were purposively selected. Dietary intake and anthropometric measurements were assessed using standardized protocols. Content validity was evaluated by a panel of ten experts through qualitative review and quantitative assessment through Lawshe’s Content Validity Ratio(CVR).

**Results:**

Adequacy components assessed the intake of cereals and grains, fruits, vegetables, dairy, total protein-rich foods, and micronutrients. Moderation components evaluated consumption of processed meat, processed fruit products, roots and tubers, cooking oil, solid fats, and added sugars. Scoring for adequacy and moderation components was based on an energy–density approach to account for variations in total energy intake of athletes from different sports. For adequacy components, scores ranged from zero(no intake) to maximum points (when intake met or exceeded the recommended cut-off), with intermediate values scored linearly. For moderation components, maximum points were assigned for intake at or below the cut-off, and zero points were assigned for intake at or above the threshold, with linear scoring in between. Thresholds for moderation components were determined based on the 85th percentile of intake observed in the study population. The ratio component was the ratio of whole grains to total grains intake. A ratio at or below the threshold ( ≤ 0.5) was assigned zero points. Behavioural components included abstinence from alcohol, adequate fluid intake, and consumption of pre- and post-training meals, which were assessed using a Likert scale. CVR for DQI-Athletes was 0.9.

**Conclusions:**

DQI-Athletes integrates adequacy, moderation, ratio, and behavioural components to evaluate the overall diet quality of athletes of different sports. High CVR confirmed the content validity of the tool.

**Supplementary Information:**

The online version contains supplementary material available at https://doi.org/10.1186/s40795-026-01282-8.

## Introduction

Sports nutrition plays a crucial role as athletic performance tends to decline before visible signs of nutrient deficiency appear [1]. Given that dietary strategies directly influence athletic performance [2], appropriate dietary assessment tools are essential for accurately evaluating athletes’ food intake and informing nutrition planning. However, assessing the diet of athletes can be challenging due to the complexity of accurately recording intake of frequent meals and supplements, the extensive analysis required to estimate both macronutrient and micronutrient intake, the lack of pre-tested dietary assessment questionnaire tailored for athletes of different sports, and the frequent long-distance travel undertaken by athletes for training and competitions [3–7]. Consequently, this may result in under-reporting and infrequent monitoring of dietary intake among athletes. Recent review paper has reported that the dietary intake of Indian athletes is insufficient, with lower intake of both macronutrients and micronutrients compared to the recommended values [8]. Although most studies emphasize meeting recommended macronutrient and micronutrient intakes, evaluating nutrient intake alone offers an incomplete picture of overall diet quality, since nutrients are consumed in combination and interact within the diet [9, 10]. Diet Quality Index (DQI) is a multi-dimensional dietary assessment tool that scores an individual’s eating patterns based on the extent to which they align with established dietary recommendations [11]. A higher DQI score is associated with improved nutrient intake and better health outcomes [11]. Diet quality indices are generally constructed around four major aspects: adequacy, moderation, balance, and variety, which collectively assess sufficient intake of health-promoting foods, restriction of foods detrimental to health, appropriate nutrient distribution, and dietary diversity [12].

Energy requirements of Indian athletes are higher than general population. Endurance events athletes participating in events such as marathon running, long-distance walking, road cycling, rowing, middle- and long-distance swimming (200 m and above) have high energy demands, with estimated energy requirements of approximately 80 kcal/kg/day. In this group, carbohydrates, protein, and fat contribute approximately 58%, 14%, and 28% of total energy intake, respectively, to support sustained aerobic performance and recovery. Power athletes in super heavy and higher weight categories, including those competing in weightlifting, wrestling, boxing, judo, and throwing disciplines, require approximately 70 kcal/kg/day to sustain substantial muscle mass and high-intensity anaerobic performance. For super heavy category power athletes, carbohydrates, protein, and fat contribute approximately 61%, 14%, and 25% of total energy intake, respectively, whereas in higher weight category power athletes, the distribution is approximately 54%, 15%, and 31%, respectively. A similar caloric demand (~ 70 kcal/kg/day) is observed among athletes of team events, racquet sports and power events of middle weight categories such as hockey, football, volleyball, basketball, handball, javelin, jumpers, sprint running, track cycling, swimming (below 200 m), water polo, tennis, badminton, middle weight categories of power events such as boxing, wrestling, weightlifting, and judo who engage in both strength and endurance activities. In this group, carbohydrates, protein, and fat contribute approximately 54%, 15%, and 31% of total energy intake. Light weight events category athletes, including those participating in gymnastics, table tennis, yachting, and lightweight combat sports, have comparatively lower requirements at approximately 60 kcal/kg/day, reflecting smaller body size and reduced absolute energy output. In this group, carbohydrates, protein, and fat contribute approximately 54%, 16%, and 30% of total energy intake. Skill sport athletes, such as those competing in shooting, archery, and equestrian sports, have the lowest energy requirements among athletic populations, estimated at approximately 50 kcal/kg/day, owing to lower physical workloads and a greater emphasis on neuromuscular precision. In this group, carbohydrates, protein, and fat contribute approximately 54%, 16%, and 30% of total energy intake [1]. Primary carbohydrate food sources include cereals and grains, starchy vegetables, and fruits. During periods of high training intensity or prolonged exertion, table sugar and jam are incorporated to facilitate rapid glycogen replenishment. Protein-rich food sources include pulses and legumes, green leafy vegetables, eggs, meat, and dairy. Athletes often use protein supplements to meet increased protein requirements, especially when dietary intake alone is inadequate. Avoiding trans fats and restricting saturated fat-rich foods are crucial for athletes’ cardiovascular health. Dietary fiber intake is also tailored to energy demands, with a healthy amount of dietary fiber varying between 25–48 g/day, typically corresponding to a total daily energy intake between 3000 and 7000 kcal. Adequate dietary fiber is critical for gastrointestinal function, glycemic control, and overall diet quality in athletes [1].

Iron and calcium are the most extensively studied micronutrients in sports nutrition. Iron deficiency is common in endurance athletes due to exercise-induced hemolysis, gastrointestinal blood loss, and inflammation associated with prolonged training and repeated ground contact [13–16]. Inadequate calcium intake can increase the risk of stress fractures and other skeletal injuries, particularly important for athletes involved in high-impact or weight-bearing sports [17]. Additionally, athletes may experience small but cumulative losses of iron and calcium through sweat, particularly during prolonged or high-intensity training in hot environments, thereby further elevating their daily micronutrient requirements. In female athletes, insufficient iron and calcium intake can contribute to hormonal imbalances and menstrual dysfunction, heightening the risk of conditions like the Female Athlete Triad or Relative Energy Deficiency in Sport (RED-S) [18–20]. Therefore, ensuring adequate iron and calcium intake is essential for preventing deficiencies and supporting optimal training, recovery, and athletic performance.

A comprehensive nutrition strategy for athletes includes well-planned hydration practices. Athletes are advised to consume approximately 200–300 mL of fluid 10–20 min before training to ensure adequate pre-training hydration. During training, fluid intake of 0.4–0.8 L per hour is recommended to maintain thermoregulation and cardiovascular function. In the post-training recovery phase, rehydration should include 1.25–1.5 L of fluid per kilogram of body weight lost, as this volume is necessary to adequately restore fluid balance. Consuming the right nutrients before training helps fuel the body, sustain energy levels, and enhance performance. Accordingly, athletes are recommended to consume 1–4 g/kg body weight of moderate GI carbohydrates 1–4 h before training, depending on exercise intensity and tolerance. Post-training nutritional recovery should be initiated promptly, ideally within 30 min of training, and include 1.0–1.5 g/kg body weight of moderate to high glycemic index carbohydrates along with high-quality protein to stimulate glycogen resynthesis, muscle protein synthesis, and tissue repair [21–24].

Given that athletes have higher nutritional requirements than the non-athletic population, a diet quality index can serve as a useful dietary assessment tool for assessing overall diet quality of athletes [5]. As dietary recommendations differ between sports, uniform assessment criteria may not accurately reflect the diet quality of athletes across various sport disciplines and categories. Applying the same dietary recommendations to super heavy weight and lightweight category power athletes or skill sport and power athletes can result in inaccurate scoring, similar to the limitations observed when using population-based indices for athletic populations. Therefore, there is a need to develop a Diet Quality Index for Athletes (DQI-Athletes) tailored to athletes of different sports and establish its content validity through expert evaluation for the Indian athletic population.

## Methodology

The Diet Quality Index for Athletes (DQI-Athletes) scoring framework ranged from 0 to 100 points, with component types categorized into adequacy, moderation, ratio, and dietary behaviours. A total of 108 athletes (aged ≥ 18 years, 52 females) training in various sports in Mumbai (India) were purposively selected. Dietary intake and anthropometric measurements were assessed for all participants. Content validity was evaluated by a panel of ten subject-matter experts using both qualitative review and quantitative assessment through Lawshe’s Content Validity Ratio (CVR) method. The study was conducted between 2020 and 2023.

### Development of Diet Quality Index for Athletes (DQI-Athletes)

The selected items of the DQI-Athletes were food groups, micronutrients, and dietary behaviours. The International Life Science Institute, in collaboration with the National Institute of Nutrition and the Sports Authority of India, has provided nutrition guidelines, recommendations, and sample diets for athletes across various sports [1]. Food items contributing to total energy intake across different categories of sports were identified from these guidelines and incorporated into the index framework to ensure representation of recommended dietary patterns.

### Participants and assessment methods

A total of 108 athletes (≥ 18 years; 52 females) were enrolled from training centres in Mumbai, India, including skill sports (n = 30), lightweight power sports (n = 30), team sports (n = 30), and endurance sports (n = 18). This exceeds the minimum recommended sample size of 100 for dietary assessment validation studies [5]. Data were collected to determine the scoring criteria for moderation components and to evaluate the suitability of the behavioural components and their scoring criteria within the index. Informed consent was obtained. Participants were purposively selected according to the inclusion criteria. Eligible athletes were men and women aged 18 years and older who competed at a level higher than the state level, had trained consistently for at least 24 months, and engaged in training for a minimum of four hours per day, six days per week. These criteria were consistent with those used to define elite athletes [25]. Participants excluded from the study were exercisers, para-athletes, and athletes during competitive and off-season periods. Additional exclusions included those undergoing weight-loss or gain regimens, following special diets, or with ongoing medical conditions. Dietary intake was assessed using a three-day 24-h dietary recall using the standardized Multiple-Pass Method [26]. Dietary intake was assessed over three non-consecutive days of a week, comprising two weekdays and one weekend day. The weekdays represented training days, while the weekend day corresponded to either a rest day or the day with the lowest training load during the week. Portion sizes were estimated using standard household measures. The nutritional composition of the reported foods was computed using the Nutritive Value of Indian Foods database [27]. To evaluate the participants’ habitual dietary patterns, a food frequency questionnaire (FFQ) was utilized. The FFQ included a comprehensive list of commonly consumed food items, and participants reported their usual frequency of intake across predefined categories, ranging from daily to never, with intermediate weekly and monthly frequencies. Alcohol intake was evaluated over the past year, whereas fluid intake and pre- and post-training meal consumption were assessed over the past month (typically a training mesocycle) using a food frequency questionnaire (FFQ). Anthropometric measurements were obtained using standardized procedures. Stature (cm) was measured with a non-stretchable fiberglass measuring tape, and body weight (kg) was assessed using a body composition monitor (Karada Scan, model HBF-362, Omron, Osaka, Japan).

### Scoring of DQI-Athletes

**Fig. 1:**
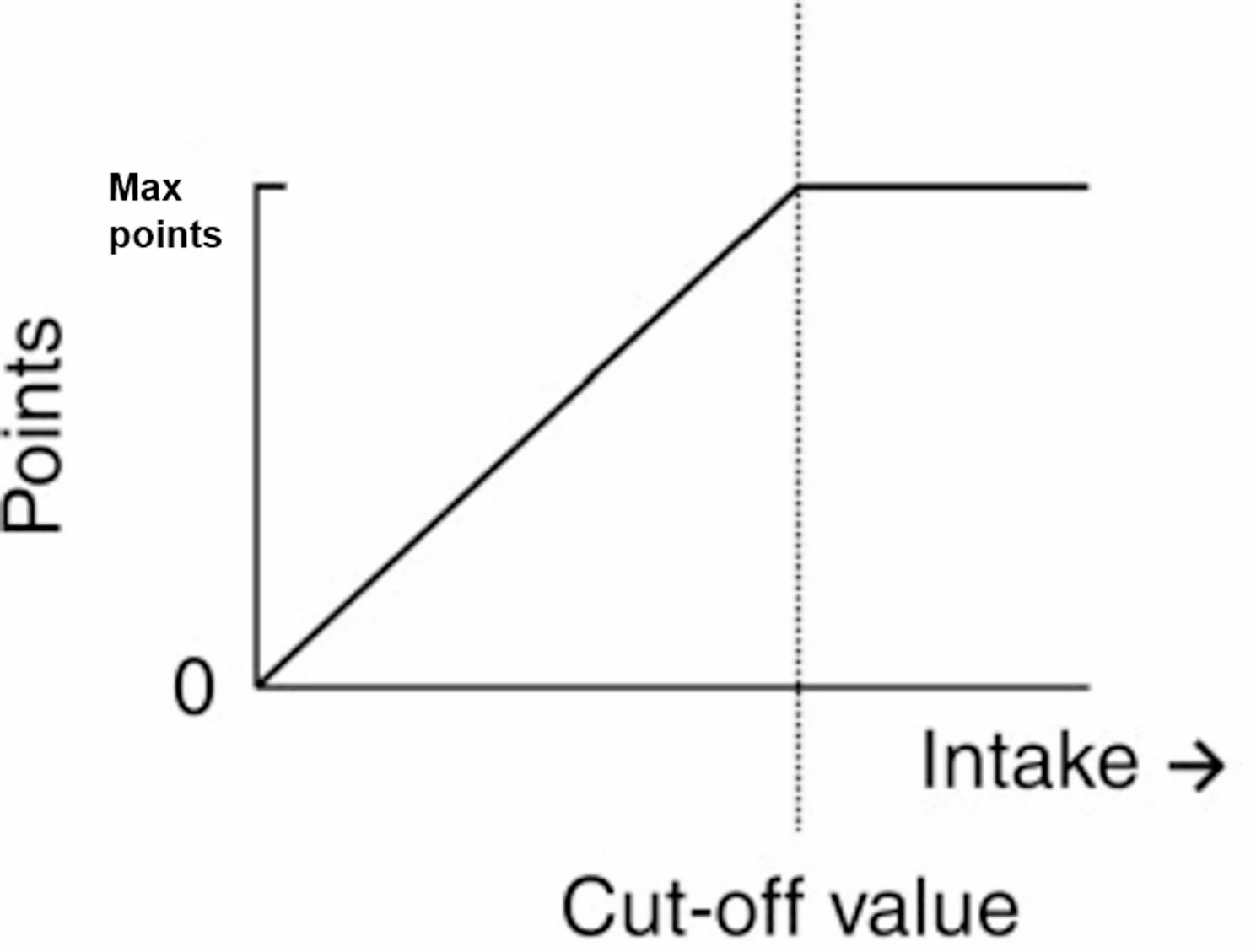
Relationship between points and adequacy component intake [28]

The total index score ranged from 0 to 100 points. Each component had a maximum score of 10 points, while subcomponents had 5 points. Scoring for adequacy and moderation components was based on an energy–density approach to account for variations in total energy intake of athletes of different sports. For the adequacy component, zero points were assigned when a food group was not consumed, while full points were awarded when the consumption amount was equal to or higher than the cut-off value, as shown in Fig. 1.The cut-off value corresponds to the recommended intake for each component. Scores between no consumption and the cut-off value were calculated linearly [28], using the following formula (1) for calculation:

1 $$Score\ for\ adequacy\ component\ = (reported\ intake / cut-off\ value) \times maximum\ score$$

**Fig. 2:**
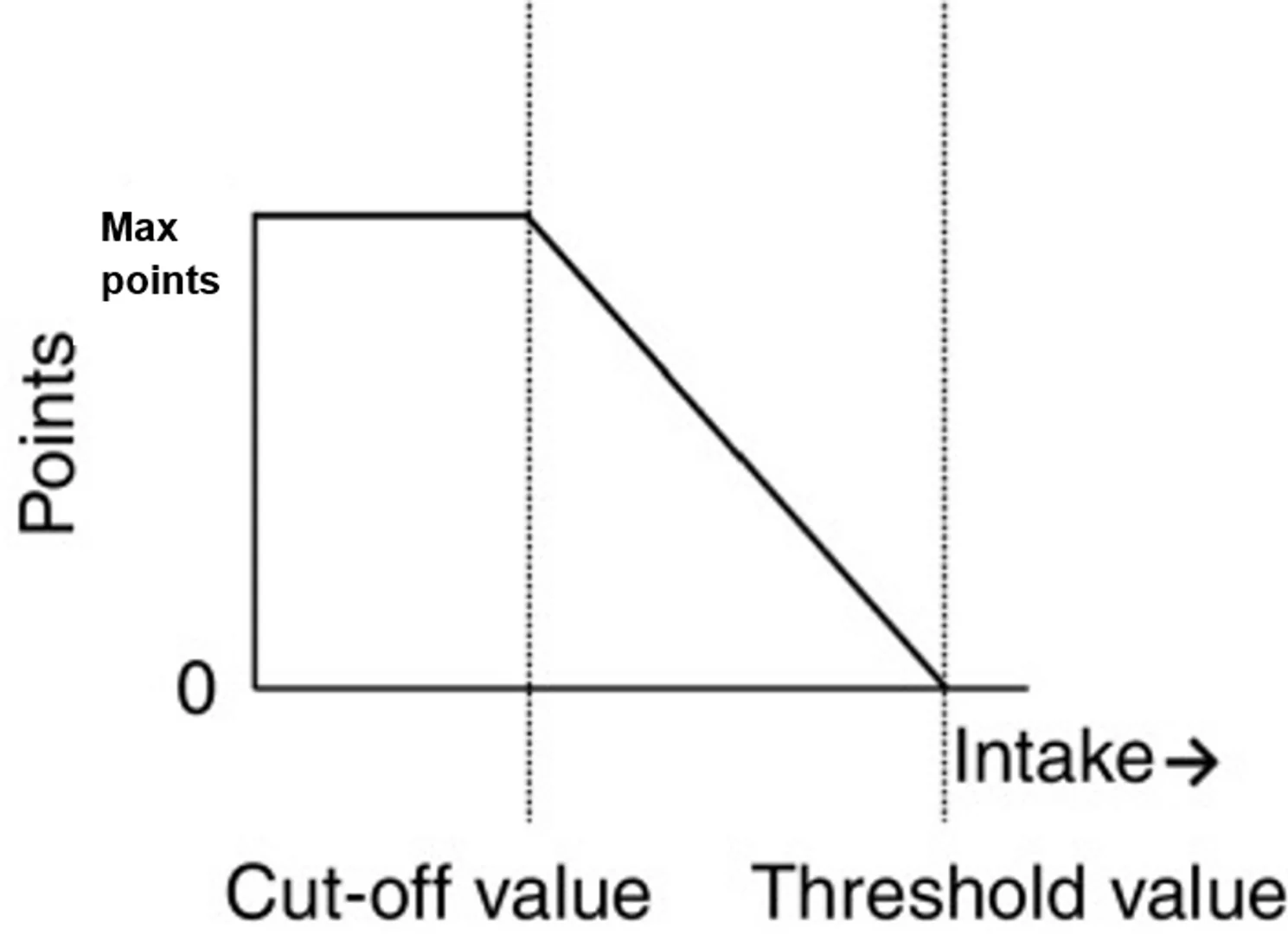
Relationship between points and the moderation component intake [28]

For the moderation component, zero points were given if intake was equal to or above the threshold value, and maximum points were assigned if intake was equal to or below the cut-off value, as shown in Fig. 2. Scores between the cut-off and threshold values were calculated linearly [28], using the following formula (2).

2 $$Score\ of\ moderation\ component\ = (threshold\ value\ -\ reported\ intake) / (threshold\ value\ -\ cut-off\ value) \times maximum\ score$$

Furthermore, the criteria for the minimum score for moderation components were derived based on the 85th percentile of the study population intake. The 85th percentiles of the study population were used in several dietary indices to establish minimum points or threshold values for moderation components, ensuring that excessive intake of certain foods is discouraged. Some well-established diet quality indices have similarly adopted this approach to define the threshold values of moderation components [29–34]. The data was analyzed in Microsoft Excel. The 85th percentile values for moderation components were not calculated for athletes in the superheavy and higher-weight power categories due to the limited representation of participants in these groups in the present study.

**Fig. 3:**
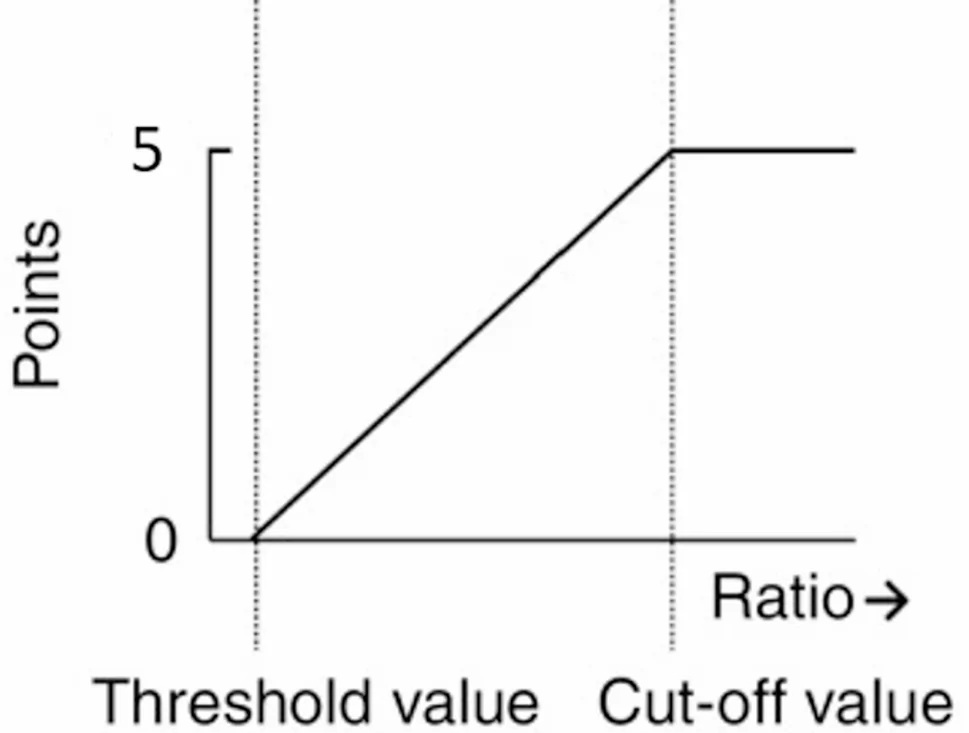
Relationship between points and ratio component intake

For the ratio component, cut-off and threshold values were established based on calculated ratios rather than absolute intake. A maximum score of five was assigned when the ratio was equal to the cut-off value, while a minimum score of zero was given if the ratio was equal to or fell below the threshold value. For ratios falling between the cut-off and threshold values, the score was determined by dividing the difference between the observed value and the threshold value by the difference between the cut-off and threshold values as shown in Fig. 3. The observed value represented the proportion of whole-grain intake relative to total grain intake and was calculated by dividing whole-grain intake by total grain intake. Scores between the cut-off and threshold values were calculated linearly using formula (3).

3 $$Score\ of\ ratio\ component\ =\ (observed\ value\ -\ threshold\ value) / (cut-off\ value\ -\ threshold\ value) \times maximum\ score$$

Behavioural components were evaluated using a Likert scale.

### Content validity

Content validity refers to the extent to which a measurement tool adequately covers all aspects of the construct it is intended to assess [6]. Content validity was evaluated by a panel of ten subject-matter experts using both qualitative review and quantitative assessment through Lawshe’s Content Validity Ratio (CVR) method. One of the most widely used methods for quantifying content validity is Lawshe’s Content Validity Ratio [35]. The CVR is a statistical technique that evaluates whether individual items in an instrument are essential for measuring the intended content domain, based on expert judgment [36].

### Identification of an expert panel

Nutritionists with a minimum of five years of experience working with athletes, as well as researchers with prior experience in developing diet quality indices, were purposively selected to validate the content of the diet quality index. Informed consent was obtained.

The determination of the appropriate number of experts for content validity assessment has traditionally been somewhat arbitrary. However, it is generally recommended to include at least five experts to ensure a reasonable level of control over chance agreement. While there is no established upper limit for the number of experts, panels rarely exceed ten members, as the probability of chance agreement decreases with an increasing number of experts [37]*.* Ten experts from India were selected.

### Content validity of DQI-Athletes by expert panel

Qualitatively, the expert panel reviewed each item for clarity, relevance and the scoring format to ensure consistency and comprehensibility. The quantitative method involved evaluating expert agreement on the essentiality of each item using Lawshe’s Content Validity Ratio. Experts rated each item on a three-point scale: 1 = Not necessary, 2 = Useful but not essential, and 3 = Essential. The formula for content validity ratio: CVR = (Ne-N/2)/(N/2), where Ne is the number of panelists rating an item as ‘Essential,’ and N is the total number of panelists. The resulting CVR values were interpreted according to the Lawshe Table to identify items that were critical for accurately representing the construct. This systematic approach ensured that the index included only items with strong content validity, thereby maintaining methodological rigor [37]. The data was analyzed in Microsoft Excel.

## Results

### Components of DQI-Athletes

The tool comprised four types of components: adequacy, moderation, ratio, and dietary behaviours. The adequacy components included the intake of cereals and grains, fruits, green leafy and other vegetables, milk and milk products, total protein-rich foods, and key micronutrients such as calcium and iron. The ratio component consisted of the ratio of whole grains to total grains intake. The moderation components included the intake of roots and tubers, processed fruit products, processed meat, added sugars, solid fats, and cooking oil. The behavioural components included abstinence from alcoholic drinks, adequate fluid intake, and consumption of pre- and post-training meals. In total, the tool comprised ten components. Table 1 presents the components and scoring criteria of DQI-Athletes.

**Table 1:** Components and scoring criteria of DQI-Athletes

Sr.No.	**Components**	**Component type**	**Maximum score**	**Minimum score**
1	Cereals and grains	Adequacy	5	0
	Whole grains: Total grains	Ratio	5	0
2	Fruits	Adequacy	5	0
	Processed fruit products	Moderation	5	0
3	Green leafy and other vegetables	Adequacy	5	0
	Roots and tubers	Moderation	5	0
4	Milk and milk products	Adequacy	10	0
5	Total protein-rich foods	Adequacy	5	0
	Processed meat	Moderation	5	0
6	Calcium	Adequacy	5	0
	Iron	Adequacy	5	0
7	Solid fats	Moderation	5	0
	Cooking oil	Moderation	5	0
8	Added sugars	Moderation	10	0
9	Abstinence from alcoholic drinks	Behavioural	5	0
	Adequate fluid intake	Behavioural	5	0
10	Pre-training meal consumption	Behavioural	5	0
	Post-training meal consumption	Behavioural	5	0
	Total Score		100	0

### Adequacy components

Adequacy components included the intake of major food groups, including cereals and grains, fruits, green leafy and other vegetables, milk and milk products, total protein-rich foods, and micronutrients. Recommendations for the total intake of various food groups for athletes across different sports disciplines and weight categories were specified in the guidelines [1]. Exclusion of micronutrient adequacy from a diet quality index could overlook critical nutrient deficiencies, particularly in athletes who may have elevated requirements [2, 38]. For athletes, it is crucial to meet 100% of the Estimated Average Requirement (EAR) for iron and calcium. The EAR for calcium is 800 mg/day for both male and female, whereas the EAR for iron is 11 mg/day for males and 15 mg/day for females [22]. Athletes meeting 100% of EAR of these micronutrients were assigned full scores. Approximately three cups (750 ml) of milk provide close to the EAR for calcium. Regarding iron, three small servings (a total of 100–120 g) of chicken typically provide approximately 1–1.5 mg of iron. The use of such food-exchange checklist facilitates rapid dietary assessment, particularly for micronutrients that are frequently overlooked. The remaining adequacy components are presented in Table 2.

**Table 2:** Scoring criteria for adequacy components of DQI-Athletes

	**Power events of super heavy category**	**Power events of higher weight category**	**Endurance events**	**Power events of middle weight category, Team events and Racquet sports**	**Events of light weight category**	**Skill sports**
**Cereals and grains**						
Total amount of cereals and grains (g)	890	730	630	550	400	350
Energy (kcal)/1000	7	6.155	5.32	4.56	3.7	3
Cereals and grains (g)/1000 kcal (Score ‘5’)	127	119	118	121	108	117
**Fruits**						
Total amount of fruits (g)	350	300	200	150	150	150
Energy (kcal)/1000	7	6.155	5.32	4.56	3.7	3
Fruits (g)/1000 kcal (Score ‘5’)	50	49	38	33	41	50
**Green leafy and other vegetables**						
Total amount of green leafy and other vegetables (g)	450	360	350	350	350	300
Energy (kcal)/1000	7	6.155	5.32	4.56	3.7	3
Green leafy and other vegetables (g)/1000 kcal (Score ‘5’)	64	58	66	77	95	100
**Milk and milk products**						
Total amount of milk and milk products (ml)	750	750	750	750	750	600
Energy (kcal)/1000	7	6.155	5.32	4.56	3.7	3
Milk and milk products (ml)/1000 kcal (Score ‘5’)	107	122	141	164	203	200
**Total protein-rich foods**						
Amount of pulses (g)	110	60	40	40	40	40
Amount of meat (g)	400	350	250	250	250	200
Amount of eggs (g)	200	150	150	100	50	50
Energy (kcal)/1000	7	6.155	5.32	4.56	3.7	3
Total servings/1000 kcal (Score ‘5’)^*^	2.7	2.4	2.2	2.3	2.6	2.7

^*^Divided pulses by 30 g, Divided meat by 35 g and Divided eggs by 50 g

Food exchanges considered in the dietary scoring for adequate components were based on standard portion sizes to ensure consistency across food groups. For cereals and grains, one flatbread (30 g flour) was considered equivalent to approximately 2 small phulkas, 1 slice of packaged bread (30–40 g, depending on the food label), or ½ cup of cooked rice, pasta, poha, upma, oats, or any other cooked cereals (prepared from 30 g uncooked). Biscuits were assessed based on nutrition labels; for most biscuits consumed in this study population, 4 biscuits were considered equivalent to one cereal exchange. For fruits, approximately 100 g (small size) of fresh fruit was considered equivalent to 20 g of dried fruit (for e.g., one large pitted Medjool date), based on total energy content. In the dairy category, approximately 250 ml of toned milk (1 cup) was considered interchangeable with about 200–225 ml (¾ cup) of curd/yoghurt, or 50 g of paneer/cottage cheese, depending on the type and processing of milk and milk products, based on total energy content. For green leafy and other vegetables, 1 cup of uncooked vegetables was considered equivalent to ½ cup of cooked vegetables. For total protein-rich foods, approximately ¾ cup of pulses/legumes/lentils (prepared from 30 g uncooked), 2 tablespoons of nut butter (30g), one whole egg (50 g) or two egg whites, 2-3 medium sized cooked prawns or small portion of cooked fish/chicken/lean red meat (prepared from 30-40g of uncooked), ¼ scoop of protein supplement or protein supplement providing approximately 7g protein were considered equivalent, based on protein content. Food exchange portions were expressed as approximate equivalents to account for natural variability in food composition, preparation methods, processing, and edible portion size. These standardized exchanges allowed the inclusion of diverse dietary practices while maintaining nutritional comparability. To facilitate global applicability, the tool uses grams rather than serving sizes for cut-off values for most of the components as presented in Table 2. All calculated values were rounded to the nearest integer, with fractions > 0.5 rounded up and fractions < 0.5 rounded down.

### Moderation components

Moderation components included intake of processed meat, processed fruit products, roots and tubers, added sugars, cooking oil, and solid fats. The recommendation for processed meat consumption is to limit processed meat intake to no more than 50 g (one serving) per day [39]. Processed meat consumption has been linked to increased risks of cardiovascular diseases, metabolic disorders, and certain cancers, supporting its inclusion as a moderation component in diet quality indices [40–44]. Therefore, maximum points were awarded for no consumption of processed meat, and a score of zero was given when intake was equal to or exceeded 50 g/day. The rest of the moderation components and their scoring criteria are presented in Table 3. For processed fruit products, food exchange of 1 tablespoon of jam (15g) was considered equivalent to approximately 100 ml of fruit juice, depending on the type and nutrition label, based on total energy content. For roots and tubers, ½ cup (75 g) of cooked roots and tubers was considered equivalent to ½ medium sized roasted sweet potato. For solid fats, 1½ teaspoons of butter was considered equivalent to 1 teaspoon of ghee or hydrogenated fat. Supplementary Table 1 presents scoring for adequacy and moderation components based on intake, and Supplementary Table 2 details the step-wise calculation of adequacy and moderation components for athletes of different sports type.

**Table 3:** Scoring criteria for moderation components of DQI-Athletes

	**Power events of super heavy category**	**Power events of higher weight category**	**Endurance events**	**Power events of middle weight category, Team events and Racquet sports**	**Events of light weight category**	**Skill sports**
**Processed fruit products**						
Total amount of processed fruit products (g)	40	40	40	20	20	20
Energy (kcal)/1000	7	6.155	5.32	4.56	3.7	3
Processed fruit products (g)/1000 kcal (Score ‘5’)	6	6.5	8	4	5	7
85th percentile of processed fruit products (g)/1000 kcal (Score ‘0’)			13	9	10	12
**Roots and tubers**						
Total amount of roots and tubers (g)	180	160	150	150	150	125
Energy (kcal)/1000	7	6.155	5.32	4.56	3.7	3
Roots and tubers (g)/1000 kcal (Score ‘5’)	26	26	28	33	41	42
85th percentile of roots and tubers (g)/1000 kcal (Score ‘0’)			33	38	46	47
**Added sugars**						
Total amount of added sugars (g)	80	80	80	80	60	60
Energy (kcal)/1000	7	6.155	5.32	4.56	3.7	3
Added sugars (g)/1000 kcal (Score ‘5’)	11	13	15	18	16	20
85th percentile of added sugars (g)/1000 kcal (Score ‘0’)			20	23	21	25
**Cooking oil**						
Total amount of cooking oil (ml)	75	60	50	50	50	50
Energy (kcal)/1000	7	6.155	5.32	4.56	3.7	3
Cooking oil (ml)/1000 kcal (Score ‘5’)	11	10	9	11	14	17
85th percentile of cooking oil (ml)/1000 kcal (Score ‘0’)			14	16	19	22
**Solid fats**						
Total amount of solid fats (g)	30	25	25	25	20	15
Energy (kcal)/1000	7	6.155	5.32	4.56	3.7	3
Solid fats (g)/1000 kcal (Score ‘5’)	4	4	5	5	5	5
85th percentile of solid fats (g)/1000 kcal (Score ‘0’)			10	10	10	10

### Ratio component

The only ratio component included in this index was the whole grains to total grains ratio. This ratio was calculated by dividing the intake of whole grains by total grain intake. Since refined cereal grains should be replaced with whole grains, it is recommended that more than half of total cereal grain consumption consists of whole grains [22]. Therefore, the cut-off value for this ratio was set at 1, while the threshold value was ≤ 0.5. A ratio below the threshold was assigned zero points, reinforcing the importance of incorporating whole grains into the diet for improved nutritional quality.

### Behavioural components

Behavioural components included abstinence from alcoholic drinks, adequate fluid intake, and consumption of pre-training and post-training meals. National Health Interview Survey (NHIS) classification of alcohol drinking status was used as presented in Table 4 [45]. Lifetime abstainers received the maximum score of 5 points, whereas current heavier drinkers received 0 points. Available evidence indicates a substantial gap in research examining the potential detrimental effects of alcohol consumption on recovery in athletes. Although alcohol has not been conclusively shown to impair athletic performance or recovery, this does not imply that its consumption before or after training or competition is recommended [46]. Therefore, public health guidelines were applied to this component.

**Table 4:** Classification of alcohol drinking status

Score	Alcohol drinking status
5	Lifetime abstainer—Fewer than 12 drinks in lifetime
4	Former infrequent drinker—Fewer than 12 drinks in any one year and no drinks in the past year or Former regular drinker -At least 12 drinks in any one year in lifetime but no drinks in the past year
3	Current infrequent drinker—1–11 drinks in past year
2	Current light drinker—At least 12 drinks in the past year but 3 drinks or fewer per week, on average over the past year
1	Current moderate drinker—More than 3 drinks but no more than 7 drinks per week for women and more than 3 drinks but no more than 14 drinks per week for men, on average over the past year
0	Current heavier drinker—More than 7 drinks per week for women; more than 14 drinks per week for men, on average over the past year

International sports nutrition recommendations for athletes were used for adequate fluid intake, pre- and post-training meal consumption [21–24]. Hydration practices were assessed across three periods: pre-training, during training, and post-training. Fluids such as plain water, fruit juices, isotonic sports drinks, and electrolyte-rich beverages are typically recommended and strategically planned by sports nutritionists. In the immediate pre-training window, 200–300 ml of fluid is recommended to be consumed 10–20 min before activity, as per the individual’s requirements. During training, athletes should aim to consume 0.4–0.8 L of fluid per hour, depending on training and environmental conditions. In the post-training recovery period, athletes should aim to consume 1.25–1.5 L of fluid per kilogram of body weight lost during training. Participants who followed these hydration strategies received full points, whereas those who did not follow any of the recommended strategies.

Adequate fluid intake was calculated using the following formula (4):

4 $$Adequate\ fluid\ intake\ score\ =\ (Pre-training\ fluid\ intake\ +\ During\ training\ fluid\ intake\ +\ Post-training\ fluid\ intake)/15 \times 5.$$

Fluid intake during each period was scored individually using a Likert scale (Always: 5 points; Very Frequently: 4 points; Occasionally: 3 points; Rarely: 2 points; Very Rarely: 1 point; Never: 0 points) and subsequently incorporated into the scoring formula. Total score for adequate fluid intake was measured using a Likert scale (Always: 5 points; Very Frequently: 4–4.9 points; Occasionally: 3–3.9 points; Rarely: 2–2.9 points; Very Rarely: 1–1.9 points; Never: < 1 point).

Pre- and post-training meal practices were also assessed. Ideally, a meal containing 1–4 g/kg body weight of carbohydrates should be consumed 1 to 4 h before exercise. A recovery meal or snack should ideally be consumed within 30 min post-training and include moderate to high GI carbohydrates (1.0–1.5 g/kg) along with high-quality protein to maximize recovery. Scores were measured using a Likert scale (Always: 5 points; Very Frequently: 4 points; Occasionally: 3 points; Rarely: 2 points; Very Rarely: 1 point; Never: 0 points).

Table 5 presents the Diet Quality Index for Athletes, including all the components and their scoring criteria. The criteria for the maximum score in Table 5 have been given in Table 2 and 3.

**Table 5:** Components and Scoring Criteria of Diet Quality Index for Athletes (DQI-Athletes) of different sports categories

Sr. No.	Components	Component type	Maximum score	Criteria for the maximum score	Minimum score	Criteria for the minimum score
1	Cereals and grains	Adequacy	5	E: ≥ 118 g/1000 kcal	0	0 g/1000 kcal
				T: ≥ 121 g/1000 kcal		
				LW: ≥ 108 g/1000 kcal		
				S: ≥ 117 g/1000 kcal		
	Whole grains: Total grains	Ratio	5	1	0	≤ 0.5
2	Fruits	Adequacy	5	E: ≥ 38 g/1000 kcal	0	0 g/1000 kcal
				T: ≥ 33 g/1000 kcal		
				LW: ≥ 41 g/1000 kcal		
				S: ≥ 50 g/1000 kcal		
	Processed fruit products	Moderation	5	E: ≤ 8 g/1000 kcal	0	E: ≥ 13 g/1000 kcal
				T: ≤ 4 g/1000 kcal		T: ≥ 9 g/1000 kcal
				LW: ≤ 5 g/1000 kcal		LW: ≥ 10 g/1000 kcal
				S: ≤ 7 g/1000 kcal		S: ≥ 12 g/1000 kcal
3	Green leafy and other vegetables	Adequacy	5	E: ≥ 66 g/1000 kcal	0	0 g/1000 kcal
				T: ≥ 77 g/1000 kcal		
				LW: ≥ 95 g/1000 kcal		
				S: ≥ 100 g/1000 kcal		
	Roots and tubers	Moderation	5	E: ≤ 28 g/1000 kcal	0	E: ≥ 33 g/1000 kcal
				T: ≤ 33 g/1000 kcal		T: ≥ 38 g/1000 kcal
				LW: ≤ 41 g/1000 kcal		LW: ≥ 46 g/1000 kcal
				S: ≤ 42 g/1000 kcal		S: ≥ 47 g/1000 kcal
4	Milk and milk products	Adequacy	10	E: ≥ 141 ml/1000 kcal	0	0 ml/1000 kcal
				T: ≥ 164 ml/1000 kcal		
				LW: ≥ 203 ml/1000 kcal		
				S: ≥ 200 ml/1000 kcal		
5	Total protein-rich foods	Adequacy	5	E: ≥ 2.2 servings/1000 kcal	0	0 serving/1000 kcal
				T: ≥ 2.3 servings/1000 kcal		
				LW: ≥ 2.6 servings/1000 kcal		
				S: ≥ 2.7 servings/1000 kcal		
	Processed meat	Moderation	5	0 g/day	0	≥ 50 g/day
6	Calcium	Adequacy	5	100% of EAR	0	0% of EAR
	Iron	Adequacy	5	100% of EAR	0	0% of EAR
7	Solid fats	Moderation	5	≤ 5 g/1000 kcal	0	≥ 10 g/1000 kcal
	Cooking oil	Moderation	5	E: ≤ 9 ml/1000 kcal	0	E: ≥ 14 ml/1000 kcal
				T: ≤ 11 ml/1000 kcal		T: ≥ 16 ml/1000 kcal
				LW: ≤ 14 ml/1000 kcal		LW: ≥ 19 ml/1000 kcal
				S: ≤ 17 ml/1000 kcal		S: ≥ 22 ml/1000 kcal
8	Added sugars	Moderation	10	E: ≤ 15 g/1000 kcal	0	E: ≥ 20 g/1000 kcal
				T: ≤ 18 g/1000 kcal		T: ≥ 23 g/1000 kcal
				LW: ≤ 16 g/1000 kcal		LW: ≥ 21 g/1000 kcal
				S: ≤ 20 g/1000 kcal		S: ≥ 25 g/1000 kcal
9	Abstinence from alcoholic drinks	Behavioural	5	Lifetime abstainer	0	Current heavier drinker
	Adequate fluid intake	Behavioural	5	Always	0	Never
10	Pre-training meal consumption	Behavioural	5	Always	0	Never
	Post-training meal consumption	Behavioural	5	Always	0	Never
	Total Score		100		0	

*E* Endurance events- Marathon, Long distance running, Long distance walking, Road cycling, Rowing, Middle- & Long-distance swimming (200 m and above), *T* Team events, Racquet sports & Power events of middleweight category- Hockey, Football, Volleyball, Basketball, Track Cycling, Javelin, Handball, Jumpers, Sprint running, Swimming (below 200 m), Water polo, Tennis, Badminton, middleweight category of power events (Boxing, Wrestling, Weightlifting, and Judo), *LW* Events of lightweight category: Gymnastics, Table Tennis, Power events of lightweight category and Yachting, *S* Skill sports: Shooting, Archery and Equestrian

### Content validity of DQI-Athletes

For a panel of ten experts, the minimum acceptable Content Validity Ratio is 0.62. In this study, the CVR for the index was calculated to be 0.9, indicating a strong level of agreement among the experts regarding the relevance of the items. For the qualitative review, the expert panel evaluated the wording, item order, and suitability of the scoring format to ensure clarity and consistency.

## Discussion

Development of DQI-Athletes represents an important advancement in enhancing nutritional assessment, informing targeted dietary interventions, and supporting optimal athletic performance. This study provides a comprehensive, sport-specific framework to evaluate diet quality among athletes of different sports, ensuring that the components and scoring criteria of diet quality index align with evidence-based recommendations. Previously, population-based indices have been applied to evaluate overall diet quality in athletic populations. Jürgensen et al. (2015) evaluated the diet quality of 72 team-sport athletes (37 women) using the Healthy Eating Index-Revised (HEI-R). None of the athletes met the criteria for a “healthy” diet, with 45.7% of men and 51.4% of women classified as having an “inadequate” diet. The lowest scores were observed for fruits, vegetables, whole grains, and dairy, whereas the meat, legumes, and eggs components scored the highest [47]. Jontony et al. (2020) assessed diet quality using the HEI-2015 in 129 NCAA Division 1 athletes. The mean HEI score was 71.0 ± 11.2, with women’s rowing showing the highest diet quality (73.5 ± 9.7) and men’s wrestling the lowest (56.5 ± 5.7). Athletes achieved their highest HEI component scores in total protein foods (4.6 ± 0.9), whole fruits (4.5 ± 1.0), and total fruits (4.3 ± 1.2) out of 5 points. Conversely, the lowest scores were observed for fatty acids (5.1 ± 2.8) and whole grains (6.3 ± 3.0) out of 10 points, indicating these areas as key contributors to poorer overall diet quality [48]. Werner et al. (2022) evaluated diet quality in 94 NCAA Division I athletes using a 24-h dietary recall and HEI. The mean HEI score was 59.2 ± 16.6, and only 9 athletes achieved a score ≥ 80, indicating good diet quality. No differences were observed across sex and sport. Overall, the study showed poor adherence to dietary guidelines, highlighting potential implications for athlete health and performance [49]. Barney et al. (2024) examined dietary intake and diet quality in 28 NCAA Division I cross-country athletes (14 women) over nine days of food records during the competitive season. HEI-2020 scores reflected suboptimal adherence to the Dietary Guidelines for Americans, with females showing significantly higher diet quality than males (65.3 ± 13.7 vs. 50.6 ± 10.1; p = 0.0034). Across the cohort, athletes did not meet recommended thresholds for any HEI-2020 food group component except for total protein-rich foods [50]. A recent scoping review by Dion et al. (2024) synthesized evidence from 18 studies assessing the diet quality of adult athletes using validated diet quality indices. Basketball players and gymnasts were the most frequently studied athlete groups, and many studies were gender-skewed (seven included only males and four included only females). Across the included studies, eleven different diet quality assessment tools were used, with variations of the Healthy Eating Index appearing most often (n = 7). Overall diet quality was generally suboptimal, with three studies classifying it as “poor,” twelve as “needs improvement,” and only three as “adequate.” The most consistently inadequate food groups were whole grains (n = 8), fruits (n = 5), and dairy/alternatives (n = 3). In contrast, intake of protein-rich foods was adequate in nine studies, while excessive fat intake was reported in four [51]. These findings suggest that the population-based indices can serve as a useful screening tool for identifying potential nutritional inadequacies; however, they do not incorporate components that are specific to athletes or sport-related dietary demands. In contrast, a previously developed diet quality index designed for elite athletes, with scoring criteria based on adherence to the Australian Guide to Healthy Eating along with international sports nutrition recommendations, has showed validity and reliability in capturing athlete-specific dietary components [52].

Our newly developed sport-specific tool for athletes is tailored to address the unique nutritional requirements of athletes of different sports disciplines and categories. This ensures that the nutritional evaluation is relevant and directly applicable to athletes competing in various sports. The present tool for athletes utilizes a continuous scoring approach, which is considered more sensitive and effective for detecting changes in dietary patterns. Unlike dichotomous scoring methods that categorize intake as simply adequate or inadequate, continuous scoring allows for a more nuanced assessment of diet quality. This makes it particularly valuable in both population-level surveillance and nutrition intervention research, where subtle dietary improvements or declines need to be accurately tracked over time. Secondly, the diet quality index was constructed using a density-based standard. The adoption of density standards enables the use of a uniform reference across individuals, irrespective of their total energy requirements, as accurately measuring energy needs can be challenging. The food consumption was assessed based on the standardized portion sizes. Incorporating standard portions enhances the effectiveness in accurately assessing overall diet quality. Using the 85th percentile for moderation components offers several methodological and interpretive advantages. Firstly, it aligns with the conventions used in widely accepted diet quality indices, enabling greater comparability across studies and populations. Secondly, it provides a balance between sensitivity and specificity, effectively identifying individuals with higher than recommended intakes without being overly restrictive. Additionally, in populations with moderate skewness in intake distributions, the 85th percentile can still offer adequate differentiation while maintaining statistical stability, avoiding the extremes and potential outliers associated with higher percentiles like the 90th or 95th. A high CVR value indicates that the items included in the tool are both appropriate and comprehensive in capturing the construct of diet quality, as intended. This enhances the credibility of the index and supports its suitability for assessing dietary patterns among athletes. This finding also underscores the importance of involving domain experts in the development and refinement of measurement instrument, especially when they are intended for use in specific populations with unique nutritional demands, such as athletes.

### Limitations and future studies

While the tool offers a more precise and tailored diet quality assessment for athletes of various sports, certain limitations must be acknowledged. Establishing a threshold value for the moderation component presents a challenge, as there is limited scientific consensus on the intake level that should warrant a minimum score of zero. If the threshold is set too high, resulting in a significant portion of the population receiving the lowest possible score, it may reduce the tool’s sensitivity to detect inter-individual or group variations and changes over time at the lower end of the scoring scale [32]. Due to the low participation of athletes in the super-heavy and higher-weight power categories in the western zone of India during the study period, 85th percentile values for moderation components could not be established for these groups. Para-athletes were not included in the study due to the lack of sport-specific dietary guidelines for this population. Furthermore, since the DQI uses a standardized energy–density based scoring methodology and follows standard dietary guidelines for athletes of different sports disciplines and categories, it remains applicable for assessing diet quality across varied athletic subgroups. Nonetheless, future studies should focus on validating the DQI in diverse athletic populations, including wider sport disciplines, categories and competitive levels. The conceptual framework and scoring methodology of this index can be adapted for use in other countries by incorporating region-specific dietary guidelines, cultural food patterns, and sport-specific nutritional recommendations. Development of a digital platform could significantly enhance its usability.

The 85th percentile values for moderation components among athletes in the super heavy and higher weight categories will be reported in future publications. Future studies will report construct validity and reliability or internal consistency of the tool. Evaluation of validity suggests that the DQI-Athletes was capable of distinguishing diet quality independently of total energy intake. The scree plot using principal component analysis showed the presence of seven factors with eigenvalues > 1. Recommended exemplary menus for athletes scored maximum diet quality scores. Higher scores (across quartiles) of green leafy vegetables and total protein-rich foods altogether were significantly associated with a higher amount of iron intake from the three-day 24-h dietary recall before and after energy adjustment. Higher scores (across quartiles) of milk and milk products, green leafy vegetables, and total protein-rich foods altogether were significantly associated with higher amounts of calcium intake from the three-day 24-h dietary recall before and after energy adjustment. Similar findings were observed with macronutrients, essential fatty acids, and dietary fibre. The reliability or internal consistency of the index was found to be acceptable using Cronbach’s alpha and the split-half method.

## Conclusions

DQI-Athletes integrates adequacy, moderation, ratio, and behavioural components to evaluate the overall diet quality of athletes of different sports. By considering sport-specific recommendations, DQI-Athletes provides a practical evaluation tool for assessing the overall diet quality of athletes of various sports. This study highlights the need to shift from isolated nutrient-based assessments to a more integrated approach that evaluates overall diet quality among athletes. DQI-Athletes has the potential to inform dietary practices, improve performance outcomes, and contribute to the long-term health of athletes. A high Content Validity Ratio value reflects a strong consensus among the expert panel regarding the essentiality and relevance of the index components in line with established recommendations, with an adaptable framework suitable for application in other countries using region-specific recommendations.

## Supplementary Information

Supplementary Material 1.

## Data Availability

The datasets used and analyzed during the current study are available from the corresponding author on reasonable request.

## Abbreviations

kcal: Kilocalories
g: Gram
mg: Milligram
CVR: Content Validity Ratio
DQI: Diet Quality Index
EAR: Estimated Average Requirement
FFQ: Food Frequency Questionnaire
HEI: Healthy Eating Index
HEI-R: Healthy Eating Index-Revised
NCAA: National Collegiate Athletic Association
NHIS: National Health Interview Survey
RED-S: Relative Energy Deficiency in Sport
