# Supplementary material for: Development and content validity of Diet Quality Index for Athletes (DQI-Athletes)
Source: BMC Nutr. 2026 Sep 21;12:195. doi: 10.1186/s40795-026-01282-8 (PMC13591869; doi:10.1186/s40795-026-01282-8)
Supplement: Supplementary file 1 [file 40795_2026_1282_MOESM1_ESM.docx]

**Supplementary Table 1. Scoring of adequacy and moderation components**

| SCORE | Power events of super heavy category | Power events of higher weight category | Endurance events | Power events of middleweight category, Team events and Racquet sports | Events of lightweight category | Skill sports |  |  |
| --- | --- | --- | --- | --- | --- | --- | --- | --- |
| CEREALS AND GRAINS (g/1000 kcal) | | | | | | |  |  |
| 5.0 | **≥127** | **≥119** | **≥118** | **≥121** | **≥108** | **≥117** |  |  |
| 4.9 | 124.46-126.99 | 116.62-118.99 | 115.64-117.99 | 118.58-120.99 | 105.84-107.99 | 114.66-116.99 |  |  |
| 4.8 | 121.92 | 114.24 | 113.28 | 116.16 | 103.68 | 112.32 |  |  |
| 4.7 | 119.38 | 111.86 | 110.92 | 113.74 | 101.52 | 109.98 |  |  |
| 4.6 | 116.84 | 109.48 | 108.56 | 111.32 | 99.36 | 107.64 |  |  |
| 4.5 | 114.30 | 107.10 | 106.20 | 108.90 | 97.20 | 105.30 |  |  |
| 4.4 | 111.76 | 104.72 | 103.84 | 106.48 | 95.04 | 102.96 |  |  |
| 4.3 | 109.22 | 102.34 | 101.48 | 104.06 | 92.88 | 100.62 |  |  |
| 4.2 | 106.68 | 99.96 | 99.12 | 101.64 | 90.72 | 98.28 |  |  |
| 4.1 | 104.14 | 97.58 | 96.76 | 99.22 | 88.56 | 95.94 |  |  |
| 4.0 | 101.60 | 95.20 | 94.40 | 96.80 | 86.40 | 93.60 |  |  |
| 3.9 | 99.06 | 92.82 | 92.04 | 94.38 | 84.24 | 91.26 |  |  |
| 3.8 | 96.52 | 90.44 | 89.68 | 91.96 | 82.08 | 88.92 |  |  |
| 3.7 | 93.98 | 88.06 | 87.32 | 89.54 | 79.92 | 86.58 |  |  |
| 3.6 | 91.44 | 85.68 | 84.96 | 87.12 | 77.76 | 84.24 |  |  |
| 3.5 | 88.90 | 83.30 | 82.60 | 84.70 | 75.60 | 81.90 |  |  |
| 3.4 | 86.36 | 80.92 | 80.24 | 82.28 | 73.44 | 79.56 |  |  |
| 3.3 | 83.82 | 78.54 | 77.88 | 79.86 | 71.28 | 77.22 |  |  |
| 3.2 | 81.28 | 76.16 | 75.52 | 77.44 | 69.12 | 74.88 |  |  |
| 3.1 | 78.74 | 73.78 | 73.16 | 75.02 | 66.96 | 72.54 |  |  |
| 3.0 | 76.20 | 71.40 | 70.80 | 72.60 | 64.80 | 70.20 |  |  |
| 2.9 | 73.66 | 69.02 | 68.44 | 70.18 | 62.64 | 67.86 |  |  |
| 2.8 | 71.12 | 66.64 | 66.08 | 67.76 | 60.48 | 65.52 |  |  |
| 2.7 | 68.58 | 64.26 | 63.72 | 65.34 | 58.32 | 63.18 |  |  |
| 2.6 | 66.04 | 61.88 | 61.36 | 62.92 | 56.16 | 60.84 |  |  |
| 2.5 | 63.50 | 59.50 | 59.00 | 60.50 | 54.00 | 58.50 |  |  |
| 2.4 | 60.96 | 57.12 | 56.64 | 58.08 | 51.84 | 56.16 |  |  |
| 2.3 | 58.42 | 54.74 | 54.28 | 55.66 | 49.68 | 53.82 |  |  |
| 2.2 | 55.88 | 52.36 | 51.92 | 53.24 | 47.52 | 51.48 |  |  |
| 2.1 | 53.34 | 49.98 | 49.56 | 50.82 | 45.36 | 49.14 |  |  |
| 2.0 | 50.80 | 47.60 | 47.20 | 48.40 | 43.20 | 46.80 |  |  |
| 1.9 | 48.26 | 45.22 | 44.84 | 45.98 | 41.04 | 44.46 |  |  |
| 1.8 | 45.72 | 42.84 | 42.48 | 43.56 | 38.88 | 42.12 |  |  |
| 1.7 | 43.18 | 40.46 | 40.12 | 41.14 | 36.72 | 39.78 |  |  |
| 1.6 | 40.64 | 38.08 | 37.76 | 38.72 | 34.56 | 37.44 |  |  |
| 1.5 | 38.10 | 35.70 | 35.40 | 36.30 | 32.40 | 35.10 |  |  |
| 1.4 | 35.56 | 33.32 | 33.04 | 33.88 | 30.24 | 32.76 |  |  |
| 1.3 | 33.02 | 30.94 | 30.68 | 31.46 | 28.08 | 30.42 |  |  |
| 1.2 | 30.48 | 28.56 | 28.32 | 29.04 | 25.92 | 28.08 |  |  |
| 1.1 | 27.94 | 26.18 | 25.96 | 26.62 | 23.76 | 25.74 |  |  |
| 1.0 | 25.40 | 23.80 | 23.60 | 24.20 | 21.60 | 23.40 |  |  |
| 0.9 | 22.86 | 21.42 | 21.24 | 21.78 | 19.44 | 21.06 |  |  |
| 0.8 | 20.32 | 19.04 | 18.88 | 19.36 | 17.28 | 18.72 |  |  |
| 0.7 | 17.78 | 16.66 | 16.52 | 16.94 | 15.12 | 16.38 |  |  |
| 0.6 | 15.24 | 14.28 | 14.16 | 14.52 | 12.96 | 14.04 |  |  |
| 0.5 | 12.70 | 11.90 | 11.80 | 12.10 | 10.80 | 11.70 |  |  |
| 0.4 | 10.16 | 9.52 | 9.44 | 9.68 | 8.64 | 9.36 |  |  |
| 0.3 | 7.62 | 7.14 | 7.08 | 7.26 | 6.48 | 7.02 |  |  |
| 0.2 | 5.08 | 4.76 | 4.72 | 4.84 | 4.32 | 4.68 |  |  |
| 0.1 | 2.54 | 2.38 | 2.36 | 2.42 | 2.16 | 2.34 |  |  |
| 0.0 | 0.00 | 0.00 | 0.00 | 0.00 | 0.00 | 0.00 |  |  |
| FRUITS (g/1000 kcal) | | | | | | |  |  |
| 5.0 | **≥50** | **≥49** | **≥38** | **≥33** | **≥41** | **≥50** |  |  |
| 4.9 | 49.00 | 48.02 | 37.24 | 32.34 | 40.18 | 49.00 |  |  |
| 4.8 | 48.00 | 47.04 | 36.48 | 31.68 | 39.36 | 48.00 |  |  |
| 4.7 | 47.00 | 46.06 | 35.72 | 31.02 | 38.54 | 47.00 |  |  |
| 4.6 | 46.00 | 45.08 | 34.96 | 30.36 | 37.72 | 46.00 |  |  |
| 4.5 | 45.00 | 44.10 | 34.20 | 29.70 | 36.90 | 45.00 |  |  |
| 4.4 | 44.00 | 43.12 | 33.44 | 29.04 | 36.08 | 44.00 |  |  |
| 4.3 | 43.00 | 42.14 | 32.68 | 28.38 | 35.26 | 43.00 |  |  |
| 4.2 | 42.00 | 41.16 | 31.92 | 27.72 | 34.44 | 42.00 |  |  |
| 4.1 | 41.00 | 40.18 | 31.16 | 27.06 | 33.62 | 41.00 |  |  |
| 4.0 | 40.00 | 39.20 | 30.40 | 26.40 | 32.80 | 40.00 |  |  |
| 3.9 | 39.00 | 38.22 | 29.64 | 25.74 | 31.98 | 39.00 |  |  |
| 3.8 | 38.00 | 37.24 | 28.88 | 25.08 | 31.16 | 38.00 |  |  |
| 3.7 | 37.00 | 36.26 | 28.12 | 24.42 | 30.34 | 37.00 |  |  |
| 3.6 | 36.00 | 35.28 | 27.36 | 23.76 | 29.52 | 36.00 |  |  |
| 3.5 | 35.00 | 34.30 | 26.60 | 23.10 | 28.70 | 35.00 |  |  |
| 3.4 | 34.00 | 33.32 | 25.84 | 22.44 | 27.88 | 34.00 |  |  |
| 3.3 | 33.00 | 32.34 | 25.08 | 21.78 | 27.06 | 33.00 |  |  |
| 3.2 | 32.00 | 31.36 | 24.32 | 21.12 | 26.24 | 32.00 |  |  |
| 3.1 | 31.00 | 30.38 | 23.56 | 20.46 | 25.42 | 31.00 |  |  |
| 3.0 | 30.00 | 29.40 | 22.80 | 19.80 | 24.60 | 30.00 |  |  |
| 2.9 | 29.00 | 28.42 | 22.04 | 19.14 | 23.78 | 29.00 |  |  |
| 2.8 | 28.00 | 27.44 | 21.28 | 18.48 | 22.96 | 28.00 |  |  |
| 2.7 | 27.00 | 26.46 | 20.52 | 17.82 | 22.14 | 27.00 |  |  |
| 2.6 | 26.00 | 25.48 | 19.76 | 17.16 | 21.32 | 26.00 |  |  |
| 2.5 | 25.00 | 24.50 | 19.00 | 16.50 | 20.50 | 25.00 |  |  |
| 2.4 | 24.00 | 23.52 | 18.24 | 15.84 | 19.68 | 24.00 |  |  |
| 2.3 | 23.00 | 22.54 | 17.48 | 15.18 | 18.86 | 23.00 |  |  |
| 2.2 | 22.00 | 21.56 | 16.72 | 14.52 | 18.04 | 22.00 |  |  |
| 2.1 | 21.00 | 20.58 | 15.96 | 13.86 | 17.22 | 21.00 |  |  |
| 2.0 | 20.00 | 19.60 | 15.20 | 13.20 | 16.40 | 20.00 |  |  |
| 1.9 | 19.00 | 18.62 | 14.44 | 12.54 | 15.58 | 19.00 |  |  |
| 1.8 | 18.00 | 17.64 | 13.68 | 11.88 | 14.76 | 18.00 |  |  |
| 1.7 | 17.00 | 16.66 | 12.92 | 11.22 | 13.94 | 17.00 |  |  |
| 1.6 | 16.00 | 15.68 | 12.16 | 10.56 | 13.12 | 16.00 |  |  |
| 1.5 | 15.00 | 14.70 | 11.40 | 9.90 | 12.30 | 15.00 |  |  |
| 1.4 | 14.00 | 13.72 | 10.64 | 9.24 | 11.48 | 14.00 |  |  |
| 1.3 | 13.00 | 12.74 | 9.88 | 8.58 | 10.66 | 13.00 |  |  |
| 1.2 | 12.00 | 11.76 | 9.12 | 7.92 | 9.84 | 12.00 |  |  |
| 1.1 | 11.00 | 10.78 | 8.36 | 7.26 | 9.02 | 11.00 |  |  |
| 1.0 | 10.00 | 9.80 | 7.60 | 6.60 | 8.20 | 10.00 |  |  |
| 0.9 | 9.00 | 8.82 | 6.84 | 5.94 | 7.38 | 9.00 |  |  |
| 0.8 | 8.00 | 7.84 | 6.08 | 5.28 | 6.56 | 8.00 |  |  |
| 0.7 | 7.00 | 6.86 | 5.32 | 4.62 | 5.74 | 7.00 |  |  |
| 0.6 | 6.00 | 5.88 | 4.56 | 3.96 | 4.92 | 6.00 |  |  |
| 0.5 | 5.00 | 4.90 | 3.80 | 3.30 | 4.10 | 5.00 |  |  |
| 0.4 | 4.00 | 3.92 | 3.04 | 2.64 | 3.28 | 4.00 |  |  |
| 0.3 | 3.00 | 2.94 | 2.28 | 1.98 | 2.46 | 3.00 |  |  |
| 0.2 | 2.00 | 1.96 | 1.52 | 1.32 | 1.64 | 2.00 |  |  |
| 0.1 | 1.00 | 0.98 | 0.76 | 0.66 | 0.82 | 1.00 |  |  |
| 0.0 | 0.00 | 0.00 | 0.00 | 0.00 | 0.00 | 0.00 |  |  |
| GREEN LEAFY AND OTHER VEGETABLES (g/1000 kcal) | | | | | | |  | 0 |
| 5.0 | **≥64** | **≥58** | **≥66** | **≥77** | **≥95** | **≥100** |  |  |
| 4.9 | 62.72 | 56.84 | 64.68 | 75.46 | 93.10 | 98.00 |  |  |
| 4.8 | 61.44 | 55.68 | 63.36 | 73.92 | 91.20 | 96.00 |  |  |
| 4.7 | 60.16 | 54.52 | 62.04 | 72.38 | 89.30 | 94.00 |  |  |
| 4.6 | 58.88 | 53.36 | 60.72 | 70.84 | 87.40 | 92.00 |  |  |
| 4.5 | 57.60 | 52.20 | 59.40 | 69.30 | 85.50 | 90.00 |  |  |
| 4.4 | 56.32 | 51.04 | 58.08 | 67.76 | 83.60 | 88.00 |  |  |
| 4.3 | 55.04 | 49.88 | 56.76 | 66.22 | 81.70 | 86.00 |  |  |
| 4.2 | 53.76 | 48.72 | 55.44 | 64.68 | 79.80 | 84.00 |  |  |
| 4.1 | 52.48 | 47.56 | 54.12 | 63.14 | 77.90 | 82.00 |  |  |
| 4.0 | 51.20 | 46.40 | 52.80 | 61.60 | 76.00 | 80.00 |  |  |
| 3.9 | 49.92 | 45.24 | 51.48 | 60.06 | 74.10 | 78.00 |  |  |
| 3.8 | 48.64 | 44.08 | 50.16 | 58.52 | 72.20 | 76.00 |  |  |
| 3.7 | 47.36 | 42.92 | 48.84 | 56.98 | 70.30 | 74.00 |  |  |
| 3.6 | 46.08 | 41.76 | 47.52 | 55.44 | 68.40 | 72.00 |  |  |
| 3.5 | 44.80 | 40.60 | 46.20 | 53.90 | 66.50 | 70.00 |  |  |
| 3.4 | 43.52 | 39.44 | 44.88 | 52.36 | 64.60 | 68.00 |  |  |
| 3.3 | 42.24 | 38.28 | 43.56 | 50.82 | 62.70 | 66.00 |  |  |
| 3.2 | 40.96 | 37.12 | 42.24 | 49.28 | 60.80 | 64.00 |  |  |
| 3.1 | 39.68 | 35.96 | 40.92 | 47.74 | 58.90 | 62.00 |  |  |
| 3.0 | 38.40 | 34.80 | 39.60 | 46.20 | 57.00 | 60.00 |  |  |
| 2.9 | 37.12 | 33.64 | 38.28 | 44.66 | 55.10 | 58.00 |  |  |
| 2.8 | 35.84 | 32.48 | 36.96 | 43.12 | 53.20 | 56.00 |  |  |
| 2.7 | 34.56 | 31.32 | 35.64 | 41.58 | 51.30 | 54.00 |  |  |
| 2.6 | 33.28 | 30.16 | 34.32 | 40.04 | 49.40 | 52.00 |  |  |
| 2.5 | 32.00 | 29.00 | 33.00 | 38.50 | 47.50 | 50.00 |  |  |
| 2.4 | 30.72 | 27.84 | 31.68 | 36.96 | 45.60 | 48.00 |  |  |
| 2.3 | 29.44 | 26.68 | 30.36 | 35.42 | 43.70 | 46.00 |  |  |
| 2.2 | 28.16 | 25.52 | 29.04 | 33.88 | 41.80 | 44.00 |  |  |
| 2.1 | 26.88 | 24.36 | 27.72 | 32.34 | 39.90 | 42.00 |  |  |
| 2.0 | 25.60 | 23.20 | 26.40 | 30.80 | 38.00 | 40.00 |  |  |
| 1.9 | 24.32 | 22.04 | 25.08 | 29.26 | 36.10 | 38.00 |  |  |
| 1.8 | 23.04 | 20.88 | 23.76 | 27.72 | 34.20 | 36.00 |  |  |
| 1.7 | 21.76 | 19.72 | 22.44 | 26.18 | 32.30 | 34.00 |  |  |
| 1.6 | 20.48 | 18.56 | 21.12 | 24.64 | 30.40 | 32.00 |  |  |
| 1.5 | 19.20 | 17.40 | 19.80 | 23.10 | 28.50 | 30.00 |  |  |
| 1.4 | 17.92 | 16.24 | 18.48 | 21.56 | 26.60 | 28.00 |  |  |
| 1.3 | 16.64 | 15.08 | 17.16 | 20.02 | 24.70 | 26.00 |  |  |
| 1.2 | 15.36 | 13.92 | 15.84 | 18.48 | 22.80 | 24.00 |  |  |
| 1.1 | 14.08 | 12.76 | 14.52 | 16.94 | 20.90 | 22.00 |  |  |
| 1.0 | 12.80 | 11.60 | 13.20 | 15.40 | 19.00 | 20.00 |  |  |
| 0.9 | 11.52 | 10.44 | 11.88 | 13.86 | 17.10 | 18.00 |  |  |
| 0.8 | 10.24 | 9.28 | 10.56 | 12.32 | 15.20 | 16.00 |  |  |
| 0.7 | 8.96 | 8.12 | 9.24 | 10.78 | 13.30 | 14.00 |  |  |
| 0.6 | 7.68 | 6.96 | 7.92 | 9.24 | 11.40 | 12.00 |  |  |
| 0.5 | 6.40 | 5.80 | 6.60 | 7.70 | 9.50 | 10.00 |  |  |
| 0.4 | 5.12 | 4.64 | 5.28 | 6.16 | 7.60 | 8.00 |  |  |
| 0.3 | 3.84 | 3.48 | 3.96 | 4.62 | 5.70 | 6.00 |  |  |
| 0.2 | 2.56 | 2.32 | 2.64 | 3.08 | 3.80 | 4.00 |  |  |
| 0.1 | 1.28 | 1.16 | 1.32 | 1.54 | 1.90 | 2.00 |  |  |
| 0.0 | 0.00 | 0.00 | 0.00 | 0.00 | 0.00 | 0.00 |  |  |
| MILK AND MILK PRODUCTS (ml/1000 kcal) | | | | | | |  |  |
| 5.0 | **≥107** | **≥121** | **≥141** | **≥164** | **≥203** | **≥200** |  |  |
| 4.9 | 104.86 | 118.58 | 138.18 | 160.72 | 198.94 | 196.00 |  |  |
| 4.8 | 102.72 | 116.16 | 135.36 | 157.44 | 194.88 | 192.00 |  |  |
| 4.7 | 100.58 | 113.74 | 132.54 | 154.16 | 190.82 | 188.00 |  |  |
| 4.6 | 98.44 | 111.32 | 129.72 | 150.88 | 186.76 | 184.00 |  |  |
| 4.5 | 96.30 | 108.90 | 126.90 | 147.60 | 182.70 | 180.00 |  |  |
| 4.4 | 94.16 | 106.48 | 124.08 | 144.32 | 178.64 | 176.00 |  |  |
| 4.3 | 92.02 | 104.06 | 121.26 | 141.04 | 174.58 | 172.00 |  |  |
| 4.2 | 89.88 | 101.64 | 118.44 | 137.76 | 170.52 | 168.00 |  |  |
| 4.1 | 87.74 | 99.22 | 115.62 | 134.48 | 166.46 | 164.00 |  |  |
| 4.0 | 85.60 | 96.80 | 112.80 | 131.20 | 162.40 | 160.00 |  |  |
| 3.9 | 83.46 | 94.38 | 109.98 | 127.92 | 158.34 | 156.00 |  |  |
| 3.8 | 81.32 | 91.96 | 107.16 | 124.64 | 154.28 | 152.00 |  |  |
| 3.7 | 79.18 | 89.54 | 104.34 | 121.36 | 150.22 | 148.00 |  |  |
| 3.6 | 77.04 | 87.12 | 101.52 | 118.08 | 146.16 | 144.00 |  |  |
| 3.5 | 74.90 | 84.70 | 98.70 | 114.80 | 142.10 | 140.00 |  |  |
| 3.4 | 72.76 | 82.28 | 95.88 | 111.52 | 138.04 | 136.00 |  |  |
| 3.3 | 70.62 | 79.86 | 93.06 | 108.24 | 133.98 | 132.00 |  |  |
| 3.2 | 68.48 | 77.44 | 90.24 | 104.96 | 129.92 | 128.00 |  |  |
| 3.1 | 66.34 | 75.02 | 87.42 | 101.68 | 125.86 | 124.00 |  |  |
| 3.0 | 64.20 | 72.60 | 84.60 | 98.40 | 121.80 | 120.00 |  |  |
| 2.9 | 62.06 | 70.18 | 81.78 | 95.12 | 117.74 | 116.00 |  |  |
| 2.8 | 59.92 | 67.76 | 78.96 | 91.84 | 113.68 | 112.00 |  |  |
| 2.7 | 57.78 | 65.34 | 76.14 | 88.56 | 109.62 | 108.00 |  |  |
| 2.6 | 55.64 | 62.92 | 73.32 | 85.28 | 105.56 | 104.00 |  |  |
| 2.5 | 53.50 | 60.50 | 70.50 | 82.00 | 101.50 | 100.00 |  |  |
| 2.4 | 51.36 | 58.08 | 67.68 | 78.72 | 97.44 | 96.00 |  |  |
| 2.3 | 49.22 | 55.66 | 64.86 | 75.44 | 93.38 | 92.00 |  |  |
| 2.2 | 47.08 | 53.24 | 62.04 | 72.16 | 89.32 | 88.00 |  |  |
| 2.1 | 44.94 | 50.82 | 59.22 | 68.88 | 85.26 | 84.00 |  |  |
| 2.0 | 42.80 | 48.40 | 56.40 | 65.60 | 81.20 | 80.00 |  |  |
| 1.9 | 40.66 | 45.98 | 53.58 | 62.32 | 77.14 | 76.00 |  |  |
| 1.8 | 38.52 | 43.56 | 50.76 | 59.04 | 73.08 | 72.00 |  |  |
| 1.7 | 36.38 | 41.14 | 47.94 | 55.76 | 69.02 | 68.00 |  |  |
| 1.6 | 34.24 | 38.72 | 45.12 | 52.48 | 64.96 | 64.00 |  |  |
| 1.5 | 32.10 | 36.30 | 42.30 | 49.20 | 60.90 | 60.00 |  |  |
| 1.4 | 29.96 | 33.88 | 39.48 | 45.92 | 56.84 | 56.00 |  |  |
| 1.3 | 27.82 | 31.46 | 36.66 | 42.64 | 52.78 | 52.00 |  |  |
| 1.2 | 25.68 | 29.04 | 33.84 | 39.36 | 48.72 | 48.00 |  |  |
| 1.1 | 23.54 | 26.62 | 31.02 | 36.08 | 44.66 | 44.00 |  |  |
| 1.0 | 21.40 | 24.20 | 28.20 | 32.80 | 40.60 | 40.00 |  |  |
| 0.9 | 19.26 | 21.78 | 25.38 | 29.52 | 36.54 | 36.00 |  |  |
| 0.8 | 17.12 | 19.36 | 22.56 | 26.24 | 32.48 | 32.00 |  |  |
| 0.7 | 14.98 | 16.94 | 19.74 | 22.96 | 28.42 | 28.00 |  |  |
| 0.6 | 12.84 | 14.52 | 16.92 | 19.68 | 24.36 | 24.00 |  |  |
| 0.5 | 10.70 | 12.10 | 14.10 | 16.40 | 20.30 | 20.00 |  |  |
| 0.4 | 8.56 | 9.68 | 11.28 | 13.12 | 16.24 | 16.00 |  |  |
| 0.3 | 6.42 | 7.26 | 8.46 | 9.84 | 12.18 | 12.00 |  |  |
| 0.2 | 4.28 | 4.84 | 5.64 | 6.56 | 8.12 | 8.00 |  |  |
| 0.1 | 2.14 | 2.42 | 2.82 | 3.28 | 4.06 | 4.00 |  |  |
| 0.0 | 0.00 | 0.00 | 0.00 | 0.00 | 0.00 | 0.00 |  |  |
| TOTAL PROTEIN-RICH FOODS (servings/1000 kcal) | | | | | | |  |  |
| 5.0 | **≥2.7** | **≥2.4** | **≥2.2** | **≥2.3** | **≥2.6** | **≥2.7** |  |  |
| 4.9 | 2.65 | 2.35 | 2.16 | 2.25 | 2.55 | 2.65 |  |  |
| 4.8 | 2.59 | 2.30 | 2.11 | 2.21 | 2.50 | 2.59 |  |  |
| 4.7 | 2.54 | 2.26 | 2.07 | 2.16 | 2.44 | 2.54 |  |  |
| 4.6 | 2.48 | 2.21 | 2.02 | 2.12 | 2.39 | 2.48 |  |  |
| 4.5 | 2.43 | 2.16 | 1.98 | 2.07 | 2.34 | 2.43 |  |  |
| 4.4 | 2.38 | 2.11 | 1.94 | 2.02 | 2.29 | 2.38 |  |  |
| 4.3 | 2.32 | 2.06 | 1.89 | 1.98 | 2.24 | 2.32 |  |  |
| 4.2 | 2.27 | 2.02 | 1.85 | 1.93 | 2.18 | 2.27 |  |  |
| 4.1 | 2.21 | 1.97 | 1.80 | 1.89 | 2.13 | 2.21 |  |  |
| 4.0 | 2.16 | 1.92 | 1.76 | 1.84 | 2.08 | 2.16 |  |  |
| 3.9 | 2.11 | 1.87 | 1.72 | 1.79 | 2.03 | 2.11 |  |  |
| 3.8 | 2.05 | 1.82 | 1.67 | 1.75 | 1.98 | 2.05 |  |  |
| 3.7 | 2.00 | 1.78 | 1.63 | 1.70 | 1.92 | 2.00 |  |  |
| 3.6 | 1.94 | 1.73 | 1.58 | 1.66 | 1.87 | 1.94 |  |  |
| 3.5 | 1.89 | 1.68 | 1.54 | 1.61 | 1.82 | 1.89 |  |  |
| 3.4 | 1.84 | 1.63 | 1.50 | 1.56 | 1.77 | 1.84 |  |  |
| 3.3 | 1.78 | 1.58 | 1.45 | 1.52 | 1.72 | 1.78 |  |  |
| 3.2 | 1.73 | 1.54 | 1.41 | 1.47 | 1.66 | 1.73 |  |  |
| 3.1 | 1.67 | 1.49 | 1.36 | 1.43 | 1.61 | 1.67 |  |  |
| 3.0 | 1.62 | 1.44 | 1.32 | 1.38 | 1.56 | 1.62 |  |  |
| 2.9 | 1.57 | 1.39 | 1.28 | 1.33 | 1.51 | 1.57 |  |  |
| 2.8 | 1.51 | 1.34 | 1.23 | 1.29 | 1.46 | 1.51 |  |  |
| 2.7 | 1.46 | 1.30 | 1.19 | 1.24 | 1.40 | 1.46 |  |  |
| 2.6 | 1.40 | 1.25 | 1.14 | 1.20 | 1.35 | 1.40 |  |  |
| 2.5 | 1.35 | 1.20 | 1.10 | 1.15 | 1.30 | 1.35 |  |  |
| 2.4 | 1.30 | 1.15 | 1.06 | 1.10 | 1.25 | 1.30 |  |  |
| 2.3 | 1.24 | 1.10 | 1.01 | 1.06 | 1.20 | 1.24 |  |  |
| 2.2 | 1.19 | 1.06 | 0.97 | 1.01 | 1.14 | 1.19 |  |  |
| 2.1 | 1.13 | 1.01 | 0.92 | 0.97 | 1.09 | 1.13 |  |  |
| 2.0 | 1.08 | 0.96 | 0.88 | 0.92 | 1.04 | 1.08 |  |  |
| 1.9 | 1.03 | 0.91 | 0.84 | 0.87 | 0.99 | 1.03 |  |  |
| 1.8 | 0.97 | 0.86 | 0.79 | 0.83 | 0.94 | 0.97 |  |  |
| 1.7 | 0.92 | 0.82 | 0.75 | 0.78 | 0.88 | 0.92 |  |  |
| 1.6 | 0.86 | 0.77 | 0.70 | 0.74 | 0.83 | 0.86 |  |  |
| 1.5 | 0.81 | 0.72 | 0.66 | 0.69 | 0.78 | 0.81 |  |  |
| 1.4 | 0.76 | 0.67 | 0.62 | 0.64 | 0.73 | 0.76 |  |  |
| 1.3 | 0.70 | 0.62 | 0.57 | 0.60 | 0.68 | 0.70 |  |  |
| 1.2 | 0.65 | 0.58 | 0.53 | 0.55 | 0.62 | 0.65 |  |  |
| 1.1 | 0.59 | 0.53 | 0.48 | 0.51 | 0.57 | 0.59 |  |  |
| 1.0 | 0.54 | 0.48 | 0.44 | 0.46 | 0.52 | 0.54 |  |  |
| 0.9 | 0.49 | 0.43 | 0.40 | 0.41 | 0.47 | 0.49 |  |  |
| 0.8 | 0.43 | 0.38 | 0.35 | 0.37 | 0.42 | 0.43 |  |  |
| 0.7 | 0.38 | 0.34 | 0.31 | 0.32 | 0.36 | 0.38 |  |  |
| 0.6 | 0.32 | 0.29 | 0.26 | 0.28 | 0.31 | 0.32 |  |  |
| 0.5 | 0.27 | 0.24 | 0.22 | 0.23 | 0.26 | 0.27 |  |  |
| 0.4 | 0.22 | 0.19 | 0.18 | 0.18 | 0.21 | 0.22 |  |  |
| 0.3 | 0.16 | 0.14 | 0.13 | 0.14 | 0.16 | 0.16 |  |  |
| 0.2 | 0.11 | 0.10 | 0.09 | 0.09 | 0.10 | 0.11 |  |  |
| 0.1 | 0.05 | 0.05 | 0.04 | 0.05 | 0.05 | 0.05 |  |  |
| 0.0 | 0.00 | 0.00 | 0.00 | 0.00 | 0.00 | 0.00 |  |  |
| MODERATION COMPONENTS | | | | | | |  |  |
| PROCESSED FRUIT PRODUCTS (g/1000 kcal) | | | | | | |  |  |
| 5.0 | **≤6** | **≤6.5** | **≤8** | **≤4** | **≤5** | **≤7** |  |  |
| 4.9 |  |  | 8.1-8.19 | 4.1-4.19 | 5.1-5.19 | 7.1-7.19 |  |  |
| 4.8 |  |  | 8.2 | 4.2 | 5.2 | 7.2 |  |  |
| 4.7 |  |  | 8.3 | 4.3 | 5.3 | 7.3 |  |  |
| 4.6 |  |  | 8.4 | 4.4 | 5.4 | 7.4 |  |  |
| 4.5 |  |  | 8.5 | 4.5 | 5.5 | 7.5 |  |  |
| 4.4 |  |  | 8.6 | 4.6 | 5.6 | 7.6 |  |  |
| 4.3 |  |  | 8.7 | 4.7 | 5.7 | 7.7 |  |  |
| 4.2 |  |  | 8.8 | 4.8 | 5.8 | 7.8 |  |  |
| 4.1 |  |  | 8.9 | 4.9 | 5.9 | 7.9 |  |  |
| 4.0 |  |  | 9.0 | 5.0 | 6.0 | 8.0 |  |  |
| 3.9 |  |  | 9.1 | 5.1 | 6.1 | 8.1 |  |  |
| 3.8 |  |  | 9.2 | 5.2 | 6.2 | 8.2 |  |  |
| 3.7 |  |  | 9.3 | 5.3 | 6.3 | 8.3 |  |  |
| 3.6 |  |  | 9.4 | 5.4 | 6.4 | 8.4 |  |  |
| 3.5 |  |  | 9.5 | 5.5 | 6.5 | 8.5 |  |  |
| 3.4 |  |  | 9.6 | 5.6 | 6.6 | 8.6 |  |  |
| 3.3 |  |  | 9.7 | 5.7 | 6.7 | 8.7 |  |  |
| 3.2 |  |  | 9.8 | 5.8 | 6.8 | 8.8 |  |  |
| 3.1 |  |  | 9.9 | 5.9 | 6.9 | 8.9 |  |  |
| 3.0 |  |  | 10.0 | 6.0 | 7.0 | 9.0 |  |  |
| 2.9 |  |  | 10.1 | 6.1 | 7.1 | 9.1 |  |  |
| 2.8 |  |  | 10.2 | 6.2 | 7.2 | 9.2 |  |  |
| 2.7 |  |  | 10.3 | 6.3 | 7.3 | 9.3 |  |  |
| 2.6 |  |  | 10.4 | 6.4 | 7.4 | 9.4 |  |  |
| 2.5 |  |  | 10.5 | 6.5 | 7.5 | 9.5 |  |  |
| 2.4 |  |  | 10.6 | 6.6 | 7.6 | 9.6 |  |  |
| 2.3 |  |  | 10.7 | 6.7 | 7.7 | 9.7 |  |  |
| 2.2 |  |  | 10.8 | 6.8 | 7.8 | 9.8 |  |  |
| 2.1 |  |  | 10.9 | 6.9 | 7.9 | 9.9 |  |  |
| 2.0 |  |  | 11.0 | 7.0 | 8.0 | 10.0 |  |  |
| 1.9 |  |  | 11.1 | 7.1 | 8.1 | 10.1 |  |  |
| 1.8 |  |  | 11.2 | 7.2 | 8.2 | 10.2 |  |  |
| 1.7 |  |  | 11.3 | 7.3 | 8.3 | 10.3 |  |  |
| 1.6 |  |  | 11.4 | 7.4 | 8.4 | 10.4 |  |  |
| 1.5 |  |  | 11.5 | 7.5 | 8.5 | 10.5 |  |  |
| 1.4 |  |  | 11.6 | 7.6 | 8.6 | 10.6 |  |  |
| 1.3 |  |  | 11.7 | 7.7 | 8.7 | 10.7 |  |  |
| 1.2 |  |  | 11.8 | 7.8 | 8.8 | 10.8 |  |  |
| 1.1 |  |  | 11.9 | 7.9 | 8.9 | 10.9 |  |  |
| 1.0 |  |  | 12.0 | 8.0 | 9.0 | 11.0 |  |  |
| 0.9 |  |  | 12.1 | 8.1 | 9.1 | 11.1 |  |  |
| 0.8 |  |  | 12.2 | 8.2 | 9.2 | 11.2 |  |  |
| 0.7 |  |  | 12.3 | 8.3 | 9.3 | 11.3 |  |  |
| 0.6 |  |  | 12.4 | 8.4 | 9.4 | 11.4 |  |  |
| 0.5 |  |  | 12.5 | 8.5 | 9.5 | 11.5 |  |  |
| 0.4 |  |  | 12.6 | 8.6 | 9.6 | 11.6 |  |  |
| 0.3 |  |  | 12.7 | 8.7 | 9.7 | 11.7 |  |  |
| 0.2 |  |  | 12.8 | 8.8 | 9.8 | 11.8 |  |  |
| 0.1 |  |  | 12.9 | 8.9 | 9.9 | 11.9 |  |  |
| 0.0 |  |  | 13.0 | 9.0 | 10.0 | 12.0 |  |  |
| ROOTS AND TUBERS (g/1000 kcal) | | | | | | |  |  |
| 5.0 | **≤26** | **≤26** | **≤28** | **≤33** | **≤41** | **≤42** |  |  |
| 4.9 |  |  | 28.1 | 33.0 | 41.1 | 42.1 |  |  |
| 4.8 |  |  | 28.2 | 33.1 | 41.2 | 42.2 |  |  |
| 4.7 |  |  | 28.3 | 33.2 | 41.3 | 42.3 |  |  |
| 4.6 |  |  | 28.4 | 33.3 | 41.4 | 42.4 |  |  |
| 4.5 |  |  | 28.5 | 33.4 | 41.5 | 42.5 |  |  |
| 4.4 |  |  | 28.6 | 33.5 | 41.6 | 42.6 |  |  |
| 4.3 |  |  | 28.7 | 33.6 | 41.7 | 42.7 |  |  |
| 4.2 |  |  | 28.8 | 33.7 | 41.8 | 42.8 |  |  |
| 4.1 |  |  | 28.9 | 33.8 | 41.9 | 42.9 |  |  |
| 4.0 |  |  | 29.0 | 33.9 | 42.0 | 43.0 |  |  |
| 3.9 |  |  | 29.1 | 34.0 | 42.1 | 43.1 |  |  |
| 3.8 |  |  | 29.2 | 34.1 | 42.2 | 43.2 |  |  |
| 3.7 |  |  | 29.3 | 34.2 | 42.3 | 43.3 |  |  |
| 3.6 |  |  | 29.4 | 34.3 | 42.4 | 43.4 |  |  |
| 3.5 |  |  | 29.5 | 34.4 | 42.5 | 43.5 |  |  |
| 3.4 |  |  | 29.6 | 34.5 | 42.6 | 43.6 |  |  |
| 3.3 |  |  | 29.7 | 34.6 | 42.7 | 43.7 |  |  |
| 3.2 |  |  | 29.8 | 34.7 | 42.8 | 43.8 |  |  |
| 3.1 |  |  | 29.9 | 34.8 | 42.9 | 43.9 |  |  |
| 3.0 |  |  | 30.0 | 34.9 | 43.0 | 44.0 |  |  |
| 2.9 |  |  | 30.1 | 35.0 | 43.1 | 44.1 |  |  |
| 2.8 |  |  | 30.2 | 35.1 | 43.2 | 44.2 |  |  |
| 2.7 |  |  | 30.3 | 35.2 | 43.3 | 44.3 |  |  |
| 2.6 |  |  | 30.4 | 35.3 | 43.4 | 44.4 |  |  |
| 2.5 |  |  | 30.5 | 35.4 | 43.5 | 44.5 |  |  |
| 2.4 |  |  | 30.6 | 35.5 | 43.6 | 44.6 |  |  |
| 2.3 |  |  | 30.7 | 35.6 | 43.7 | 44.7 |  |  |
| 2.2 |  |  | 30.8 | 35.7 | 43.8 | 44.8 |  |  |
| 2.1 |  |  | 30.9 | 35.8 | 43.9 | 44.9 |  |  |
| 2.0 |  |  | 31.0 | 35.9 | 44.0 | 45.0 |  |  |
| 1.9 |  |  | 31.1 | 36.0 | 44.1 | 45.1 |  |  |
| 1.8 |  |  | 31.2 | 36.1 | 44.2 | 45.2 |  |  |
| 1.7 |  |  | 31.3 | 36.2 | 44.3 | 45.3 |  |  |
| 1.6 |  |  | 31.4 | 36.3 | 44.4 | 45.4 |  |  |
| 1.5 |  |  | 31.5 | 36.4 | 44.5 | 45.5 |  |  |
| 1.4 |  |  | 31.6 | 36.5 | 44.6 | 45.6 |  |  |
| 1.3 |  |  | 31.7 | 36.6 | 44.7 | 45.7 |  |  |
| 1.2 |  |  | 31.8 | 36.7 | 44.8 | 45.8 |  |  |
| 1.1 |  |  | 31.9 | 36.8 | 44.9 | 45.9 |  |  |
| 1.0 |  |  | 32.0 | 36.9 | 45.0 | 46.0 |  |  |
| 0.9 |  |  | 32.1 | 37.0 | 45.1 | 46.1 |  |  |
| 0.8 |  |  | 32.2 | 37.1 | 45.2 | 46.2 |  |  |
| 0.7 |  |  | 32.3 | 37.2 | 45.3 | 46.3 |  |  |
| 0.6 |  |  | 32.4 | 37.3 | 45.4 | 46.4 |  |  |
| 0.5 |  |  | 32.5 | 37.4 | 45.5 | 46.5 |  |  |
| 0.4 |  |  | 32.6 | 37.5 | 45.6 | 46.6 |  |  |
| 0.3 |  |  | 32.7 | 37.6 | 45.7 | 46.7 |  |  |
| 0.2 |  |  | 32.8 | 37.8 | 45.8 | 46.8 |  |  |
| 0.1 |  |  | 32.9 | 37.9 | 45.9 | 46.9 |  |  |
| 0.0 |  |  | 33.0 | 38.0 | 46.0 | 47.0 |  |  |
| ADDED SUGARS (g/1000 kcal) | | | | | | |  |  |
| 5.0 | **≤11** | **≤13** | **≤15** | **≤18** | **≤16** | **≤20** |  |  |
| 4.9 |  |  | 15.1 | 18.1 | 16.1 | 20.1 |  |  |
| 4.8 |  |  | 15.2 | 18.2 | 16.2 | 20.2 |  |  |
| 4.7 |  |  | 15.3 | 18.3 | 16.3 | 20.3 |  |  |
| 4.6 |  |  | 15.4 | 18.4 | 16.4 | 20.4 |  |  |
| 4.5 |  |  | 15.5 | 18.5 | 16.5 | 20.5 |  |  |
| 4.4 |  |  | 15.6 | 18.6 | 16.6 | 20.6 |  |  |
| 4.3 |  |  | 15.7 | 18.7 | 16.7 | 20.7 |  |  |
| 4.2 |  |  | 15.8 | 18.8 | 16.8 | 20.8 |  |  |
| 4.1 |  |  | 15.9 | 18.9 | 16.9 | 20.9 |  |  |
| 4.0 |  |  | 16.0 | 19.0 | 17.0 | 21.0 |  |  |
| 3.9 |  |  | 16.1 | 19.1 | 17.1 | 21.1 |  |  |
| 3.8 |  |  | 16.2 | 19.2 | 17.2 | 21.2 |  |  |
| 3.7 |  |  | 16.3 | 19.3 | 17.3 | 21.3 |  |  |
| 3.6 |  |  | 16.4 | 19.4 | 17.4 | 21.4 |  |  |
| 3.5 |  |  | 16.5 | 19.5 | 17.5 | 21.5 |  |  |
| 3.4 |  |  | 16.6 | 19.6 | 17.6 | 21.6 |  |  |
| 3.3 |  |  | 16.7 | 19.7 | 17.7 | 21.7 |  |  |
| 3.2 |  |  | 16.8 | 19.8 | 17.8 | 21.8 |  |  |
| 3.1 |  |  | 16.9 | 19.9 | 17.9 | 21.9 |  |  |
| 3.0 |  |  | 17.0 | 20.0 | 18.0 | 22.0 |  |  |
| 2.9 |  |  | 17.1 | 20.1 | 18.1 | 22.1 |  |  |
| 2.8 |  |  | 17.2 | 20.2 | 18.2 | 22.2 |  |  |
| 2.7 |  |  | 17.3 | 20.3 | 18.3 | 22.3 |  |  |
| 2.6 |  |  | 17.4 | 20.4 | 18.4 | 22.4 |  |  |
| 2.5 |  |  | 17.5 | 20.5 | 18.5 | 22.5 |  |  |
| 2.4 |  |  | 17.6 | 20.6 | 18.6 | 22.6 |  |  |
| 2.3 |  |  | 17.7 | 20.7 | 18.7 | 22.7 |  |  |
| 2.2 |  |  | 17.8 | 20.8 | 18.8 | 22.8 |  |  |
| 2.1 |  |  | 17.9 | 20.9 | 18.9 | 22.9 |  |  |
| 2.0 |  |  | 18.0 | 21.0 | 19.0 | 23.0 |  |  |
| 1.9 |  |  | 18.1 | 21.1 | 19.1 | 23.1 |  |  |
| 1.8 |  |  | 18.2 | 21.2 | 19.2 | 23.2 |  |  |
| 1.7 |  |  | 18.3 | 21.3 | 19.3 | 23.3 |  |  |
| 1.6 |  |  | 18.4 | 21.4 | 19.4 | 23.4 |  |  |
| 1.5 |  |  | 18.5 | 21.5 | 19.5 | 23.5 |  |  |
| 1.4 |  |  | 18.6 | 21.6 | 19.6 | 23.6 |  |  |
| 1.3 |  |  | 18.7 | 21.7 | 19.7 | 23.7 |  |  |
| 1.2 |  |  | 18.8 | 21.8 | 19.8 | 23.8 |  |  |
| 1.1 |  |  | 18.9 | 21.9 | 19.9 | 23.9 |  |  |
| 1.0 |  |  | 19.0 | 22.0 | 20.0 | 24.0 |  |  |
| 0.9 |  |  | 19.1 | 22.1 | 20.1 | 24.1 |  |  |
| 0.8 |  |  | 19.2 | 22.2 | 20.2 | 24.2 |  |  |
| 0.7 |  |  | 19.3 | 22.3 | 20.3 | 24.3 |  |  |
| 0.6 |  |  | 19.4 | 22.4 | 20.4 | 24.4 |  |  |
| 0.5 |  |  | 19.5 | 22.5 | 20.5 | 24.5 |  |  |
| 0.4 |  |  | 19.6 | 22.6 | 20.6 | 24.6 |  |  |
| 0.3 |  |  | 19.7 | 22.7 | 20.7 | 24.7 |  |  |
| 0.2 |  |  | 19.8 | 22.8 | 20.8 | 24.8 |  |  |
| 0.1 |  |  | 19.9 | 22.9 | 20.9 | 24.9 |  |  |
| 0.0 |  |  | 20.0 | 23.0 | 21.0 | 25.0 |  |  |
| COOKING OIL (ml/1000 kcal) | | | | | | |  |  |
| 5.0 | **≤11** | **≤10** | **≤9** | **≤11** | **≤14** | **≤17** |  |  |
| 4.9 |  |  | 9.1 | 11.1 | 14.1 | 17.1 |  |  |
| 4.8 |  |  | 9.2 | 11.2 | 14.2 | 17.2 |  |  |
| 4.7 |  |  | 9.3 | 11.3 | 14.3 | 17.3 |  |  |
| 4.6 |  |  | 9.4 | 11.4 | 14.4 | 17.4 |  |  |
| 4.5 |  |  | 9.5 | 11.5 | 14.5 | 17.5 |  |  |
| 4.4 |  |  | 9.6 | 11.6 | 14.6 | 17.6 |  |  |
| 4.3 |  |  | 9.7 | 11.7 | 14.7 | 17.7 |  |  |
| 4.2 |  |  | 9.8 | 11.8 | 14.8 | 17.8 |  |  |
| 4.1 |  |  | 9.9 | 11.9 | 14.9 | 17.9 |  |  |
| 4.0 |  |  | 10.0 | 12.0 | 15.0 | 18.0 |  |  |
| 3.9 |  |  | 10.1 | 12.1 | 15.1 | 18.1 |  |  |
| 3.8 |  |  | 10.2 | 12.2 | 15.2 | 18.2 |  |  |
| 3.7 |  |  | 10.3 | 12.3 | 15.3 | 18.3 |  |  |
| 3.6 |  |  | 10.4 | 12.4 | 15.4 | 18.4 |  |  |
| 3.5 |  |  | 10.5 | 12.5 | 15.5 | 18.5 |  |  |
| 3.4 |  |  | 10.6 | 12.6 | 15.6 | 18.6 |  |  |
| 3.3 |  |  | 10.7 | 12.7 | 15.7 | 18.7 |  |  |
| 3.2 |  |  | 10.8 | 12.8 | 15.8 | 18.8 |  |  |
| 3.1 |  |  | 10.9 | 12.9 | 15.9 | 18.9 |  |  |
| 3.0 |  |  | 11.0 | 13.0 | 16.0 | 19.0 |  |  |
| 2.9 |  |  | 11.1 | 13.1 | 16.1 | 19.1 |  |  |
| 2.8 |  |  | 11.2 | 13.2 | 16.2 | 19.2 |  |  |
| 2.7 |  |  | 11.3 | 13.3 | 16.3 | 19.3 |  |  |
| 2.6 |  |  | 11.4 | 13.4 | 16.4 | 19.4 |  |  |
| 2.5 |  |  | 11.5 | 13.5 | 16.5 | 19.5 |  |  |
| 2.4 |  |  | 11.6 | 13.6 | 16.6 | 19.6 |  |  |
| 2.3 |  |  | 11.7 | 13.7 | 16.7 | 19.7 |  |  |
| 2.2 |  |  | 11.8 | 13.8 | 16.8 | 19.8 |  |  |
| 2.1 |  |  | 11.9 | 13.9 | 16.9 | 19.9 |  |  |
| 2.0 |  |  | 12.0 | 14.0 | 17.0 | 20.0 |  |  |
| 1.9 |  |  | 12.1 | 14.1 | 17.1 | 20.1 |  |  |
| 1.8 |  |  | 12.2 | 14.2 | 17.2 | 20.2 |  |  |
| 1.7 |  |  | 12.3 | 14.3 | 17.3 | 20.3 |  |  |
| 1.6 |  |  | 12.4 | 14.4 | 17.4 | 20.4 |  |  |
| 1.5 |  |  | 12.5 | 14.5 | 17.5 | 20.5 |  |  |
| 1.4 |  |  | 12.6 | 14.6 | 17.6 | 20.6 |  |  |
| 1.3 |  |  | 12.7 | 14.7 | 17.7 | 20.7 |  |  |
| 1.2 |  |  | 12.8 | 14.8 | 17.8 | 20.8 |  |  |
| 1.1 |  |  | 12.9 | 14.9 | 17.9 | 20.9 |  |  |
| 1.0 |  |  | 13.0 | 15.0 | 18.0 | 21.0 |  |  |
| 0.9 |  |  | 13.1 | 15.1 | 18.1 | 21.1 |  |  |
| 0.8 |  |  | 13.2 | 15.2 | 18.2 | 21.2 |  |  |
| 0.7 |  |  | 13.3 | 15.3 | 18.3 | 21.3 |  |  |
| 0.6 |  |  | 13.4 | 15.4 | 18.4 | 21.4 |  |  |
| 0.5 |  |  | 13.5 | 15.5 | 18.5 | 21.5 |  |  |
| 0.4 |  |  | 13.6 | 15.6 | 18.6 | 21.6 |  |  |
| 0.3 |  |  | 13.7 | 15.7 | 18.7 | 21.7 |  |  |
| 0.2 |  |  | 13.8 | 15.8 | 18.8 | 21.8 |  |  |
| 0.1 |  |  | 13.9 | 15.9 | 18.9 | 21.9 |  |  |
| 0.0 |  |  | 14.0 | 16.0 | 19.0 | 22.0 |  |  |
| SOLID FATS (g/1000 kcal) | | | | | | |  |  |
| 5.0 | **≤4** | **≤4** | **≤5** | **≤5** | **≤5** | **≤5** |  |  |
| 4.9 |  |  | 5.1 | 5.1 | 5.1 | 5.1 |  |  |
| 4.8 |  |  | 5.2 | 5.2 | 5.2 | 5.2 |  |  |
| 4.7 |  |  | 5.3 | 5.3 | 5.3 | 5.3 |  |  |
| 4.6 |  |  | 5.4 | 5.4 | 5.4 | 5.4 |  |  |
| 4.5 |  |  | 5.5 | 5.5 | 5.5 | 5.5 |  |  |
| 4.4 |  |  | 5.6 | 5.6 | 5.6 | 5.6 |  |  |
| 4.3 |  |  | 5.7 | 5.7 | 5.7 | 5.7 |  |  |
| 4.2 |  |  | 5.8 | 5.8 | 5.8 | 5.8 |  |  |
| 4.1 |  |  | 5.9 | 5.9 | 5.9 | 5.9 |  |  |
| 4.0 |  |  | 6.0 | 6.0 | 6.0 | 6.0 |  |  |
| 3.9 |  |  | 6.1 | 6.1 | 6.1 | 6.1 |  |  |
| 3.8 |  |  | 6.2 | 6.2 | 6.2 | 6.2 |  |  |
| 3.7 |  |  | 6.3 | 6.3 | 6.3 | 6.3 |  |  |
| 3.6 |  |  | 6.4 | 6.4 | 6.4 | 6.4 |  |  |
| 3.5 |  |  | 6.5 | 6.5 | 6.5 | 6.5 |  |  |
| 3.4 |  |  | 6.6 | 6.6 | 6.6 | 6.6 |  |  |
| 3.3 |  |  | 6.7 | 6.7 | 6.7 | 6.7 |  |  |
| 3.2 |  |  | 6.8 | 6.8 | 6.8 | 6.8 |  |  |
| 3.1 |  |  | 6.9 | 6.9 | 6.9 | 6.9 |  |  |
| 3.0 |  |  | 7.0 | 7.0 | 7.0 | 7.0 |  |  |
| 2.9 |  |  | 7.1 | 7.1 | 7.1 | 7.1 |  |  |
| 2.8 |  |  | 7.2 | 7.2 | 7.2 | 7.2 |  |  |
| 2.7 |  |  | 7.3 | 7.3 | 7.3 | 7.3 |  |  |
| 2.6 |  |  | 7.4 | 7.4 | 7.4 | 7.4 |  |  |
| 2.5 |  |  | 7.5 | 7.5 | 7.5 | 7.5 |  |  |
| 2.4 |  |  | 7.6 | 7.6 | 7.6 | 7.6 |  |  |
| 2.3 |  |  | 7.7 | 7.7 | 7.7 | 7.7 |  |  |
| 2.2 |  |  | 7.8 | 7.8 | 7.8 | 7.8 |  |  |
| 2.1 |  |  | 7.9 | 7.9 | 7.9 | 7.9 |  |  |
| 2.0 |  |  | 8.0 | 8.0 | 8.0 | 8.0 |  |  |
| 1.9 |  |  | 8.1 | 8.1 | 8.1 | 8.1 |  |  |
| 1.8 |  |  | 8.2 | 8.2 | 8.2 | 8.2 |  |  |
| 1.7 |  |  | 8.3 | 8.3 | 8.3 | 8.3 |  |  |
| 1.6 |  |  | 8.4 | 8.4 | 8.4 | 8.4 |  |  |
| 1.5 |  |  | 8.5 | 8.5 | 8.5 | 8.5 |  |  |
| 1.4 |  |  | 8.6 | 8.6 | 8.6 | 8.6 |  |  |
| 1.3 |  |  | 8.7 | 8.7 | 8.7 | 8.7 |  |  |
| 1.2 |  |  | 8.8 | 8.8 | 8.8 | 8.8 |  |  |
| 1.1 |  |  | 8.9 | 8.9 | 8.9 | 8.9 |  |  |
| 1.0 |  |  | 9.0 | 9.0 | 9.0 | 9.0 |  |  |
| 0.9 |  |  | 9.1 | 9.1 | 9.1 | 9.1 |  |  |
| 0.8 |  |  | 9.2 | 9.2 | 9.2 | 9.2 |  |  |
| 0.7 |  |  | 9.3 | 9.3 | 9.3 | 9.3 |  |  |
| 0.6 |  |  | 9.4 | 9.4 | 9.4 | 9.4 |  |  |
| 0.5 |  |  | 9.5 | 9.5 | 9.5 | 9.5 |  |  |
| 0.4 |  |  | 9.6 | 9.6 | 9.6 | 9.6 |  |  |
| 0.3 |  |  | 9.7 | 9.7 | 9.7 | 9.7 |  |  |
| 0.2 |  |  | 9.8 | 9.8 | 9.8 | 9.8 |  |  |
| 0.1 |  |  | 9.9 | 9.9 | 9.9 | 9.9 |  |  |
| 0.0 |  |  | 10.0 | 10.0 | 10.0 | 10.0 |  |  |

*Intermediate score ranges can be understood in Cereals and grains under Adequacy component and Processed fruit products under Moderation component for score 4.9.*

**Power events of super-heavy/higher weight category:** Heavy weight lifting, Boxing, Wrestling, Judo, and Throwing events (hammer, shot-put, discus, etc.), **Endurance events:** Marathon, Long distance running, Long distance walking, Road cycling, Rowing, Middle- and Long-distance swimming (200 m and above.), **Power events of middleweight categories, Team events and Racquet sports:** Middleweight Categories of Power events like Boxing, Wrestling, Weightlifting, and Judo, Hockey, Football, Volleyball, Basketball, Handball, Tennis, Badminton, Javelin, Jumpers, Sprint running, Track Cycling, and Swimming (below 200 m), Water Polo, **Events of lightweight category:** Gymnastics, Table Tennis and Power events of lightweight category, and Yachting, **Skill sports:** Shooting, Archery, and Equestrian

**Supplementary Table 2. Calculation of scoring of adequacy and moderation component**

|  | Power events of super heavy category | Power events of higher weight category | Endurance events | Power events of middleweight category, Team events and Racquet sports | Events of lightweight category | Skill sports |
| --- | --- | --- | --- | --- | --- | --- |
| Recommended total energy intake (kcal/day) | 7000 | 6155 | 5320 | 4560 | 3700 | 3000 |
| Calculation of scoring of adequacy component- Cereals and Grains | | | | | | |
| Recommended intake of cereals and grains (g/day) | 890 | 730 | 630 | 550 | 400 | 350 |
| Cereals and grains (g/1000 kcal) = Recommended intake of cereals and grains (g/day) ÷ recommended total energy intake (kcal/day) × 1000  (Cut-off for maximum score) | 127 | 119 | 118 | 121 | 108 | 117 |
| Calculation of scoring of moderation component- Roots and Tubers | | | | | | |
| Recommended intake of roots and tubers (g/day) | 180 | 160 | 150 | 150 | 150 | 125 |
| Roots and tubers (g/1000 kcal) = Recommended intake of roots and tubers (g/day) ÷ recommended total energy intake (kcal/day) × 1000  (Cut-off for maximum score) | 26 | 26 | 28 | 33 | 41 | 42 |
| 85^th^ percentile of intake of roots and tubers (g/1000 kcal) (Threshold for minimum score) | - | - | 33 | 38 | 46 | 47 |

*Values with decimal parts ≥0.5 were rounded up to the nearest integer, whereas values <0.5 were rounded down.*
